# Supplementary material for: Engineering surface dipoles on mixed conducting oxides with ultra-thin oxide decoration layers
Source: Nat Commun. 2024 Feb 26;15:1730. doi: 10.1038/s41467-024-45824-9 (PMC11258326; doi:10.1038/s41467-024-45824-9)
Supplement: Supplementary file 1 — Supplementary Information [file 41467_2024_45824_MOESM1_ESM.pdf]

Supplementary Information

Engineering surface dipoles on MIEC oxides with ultra-thin decoration layers

Matthäus Siebenhofer[1,2]*, Andreas Nenning[1], Christoph Rameshan[3], Peter Blaha[4], Jürgen Fleig[1], Markus Kubicek[1]*

[1]*Institute of Chemical Technologies and Analytics, TU Wien, Vienna, Austria*

[2]*Department of Nuclear Science and Engineering, MIT, Cambridge, USA*

[3]*Chair of Physical Chemistry, Montanuniversität Leoben, Leoben, Austria*

[4]*Institute of Materials Chemistry, TU Wien, Vienna, Austria*

***Corresponding authors**: Matthäus Siebenhofer: msieben@mit.edu; Markus Kubicek: markus.kubicek@tuwien.ac.at

Supplementary Note 1: Correlations of different descriptors

To investigate the interchangeability of different descriptors and properties of oxidic decorations, we compared the ionic potential, the Smith acidity, the electronegativity and the bond ionicity of a variety of binary oxides (or their respective cations). In the following, the descriptors and their origins are briefly described:

- The *ionic potential* is defined as the ratio of ionic charge and ionic radius and has the unit e/Å[1,2]. It is a measure of the charge density at the "surface" of an ion. Low ionic potential ions tend to be large ions with low charge, such as Li^{+} or Sr^{2+}, while high ionic potential ions tend to be small, high charge ions such as Cr^{6+} or Si^{4+}. The ionic potential is frequently used to describe the solubility of minerals and ionic interactions[3,4]. In this work, we used the formal charges of cations in the oxides and the crystal radii for the correct coordination proposed by Shannon[5].

- The *Smith acidity* is a measure of an oxide's thermodynamic tendency to accept or release O^{2-} ions and is based on the formation enthalpy of an oxoacid salt from an acidic oxide and a basic oxide[6], e.g.:

$$CaO + CO_2 \rightarrow CaCO_3 \tag{1}$$

In this reaction, the CaO formally passes one O^{2-} ion to the CO_2 and leads to a compound with a Ca^{2+} and a CO_3^{2-} ion. Every oxide is then assigned a number a according to the empirical expression

$$[a(A) - a(B)]^2 = h(A,B) \tag{2}$$

where A is the acidic oxide, B the basic oxide and $h(A,B)$ the standard formation enthalpy of the oxoacid salt. Moreover, the stoichiometric equations are normalized such that exactly one O^{2-} ion is transferred in the reaction. To obtain the Smith acidity scale, $a(H_2O)$ is fixed to 0.

- The *electronegativity* of an oxide depends strongly on the electronegativity of the respective cation, which itself depends on the valence state of the ion in the compound. There have been several attempts to estimate electronegativities for cations in different valence states and to evaluate an electronegativity for a binary oxide[7–9], for the comparison in this chapter, we use the electronegativity values proposed by Matar et al.[7].

- The *bond ionicity* of an oxide describes the asymmetry of a metal-oxygen bond in an oxide compound. It is derived from the electronegativities of the oxide's constituents, so it is not surprising that it correlates well with the other metrics. In this study, bond ionicity values are taken from the work of Zhuravlev[10].

In general, the comparison shows the expected result that the here explained metrics correlate very well with each other and are similarly well suitable to describe the properties of binary oxides. Differences emerge in their ease of use when considering more complicated oxides and in particular their surfaces. There, the Smith acidity, which is based on empirical data from binary oxides (and which also considers the structure of the products of reactions between binary oxides) is potentially not the best choice to describe the effects induced by modification processes. We suspect that real decorated surfaces deviate considerably from binary oxide structures and that it is the introduced cation that is decisive for the observed changes. Here we suggest that acidity and basicity (which are intuitive concepts and therefore desirable) are better correlated to other underlying ion-specific metrics such as the ionic potential or the electronegativity, which may be better suited to tackle more complicated problems. The ionic potential also has two further advantages: i) ionic radii are listed for different oxidation states, increasing the flexibility of the descriptor, ii) its constituents can be estimated by computational approaches, facilitating the synergy of experimental and theoretical studies. An overview of the quantitative correlations of the discussed metrics is given in the following figure (deviations for the ionic potential of SiO_2 might be due to the strong covalent character of the bond and our use of the formal 4+ charge):

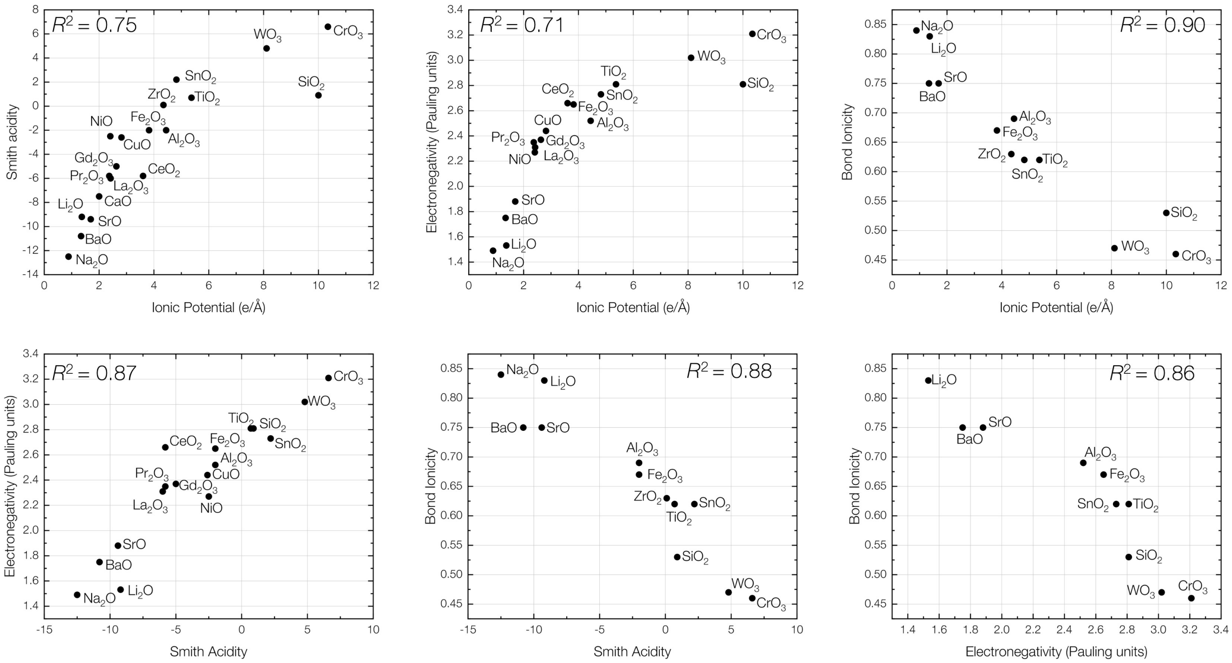

Supplementary Figure 1. Correlations of ionic potential, Smith acidity, ion electronegativity and bond ionicity. Data have been taken from several sources[5–7,10]. All metrics show good correlations with $R^2 > 0.7$, however, true linear relationships are not necessarily physically meaningful.

Supplementary Note 2: XPS details

In the following section, details of the XPS analysis for LSC thin films are shown. For XPS spectra and a detailed analysis of pristine and decorated PCO thin films please refer to a previous publication[11]. The following figure shows the XPS spectra in the low-kinetic-energy-cutoff region, the O 1*s* region and the Sr 3*d* region for a pristine, a SrO decorated and a SnO_2 decorated LSC thin film at an applied potential of 800 mV against Fe/FeO (corresponding to 10^{-6} mbar p(O_2)) at 450 °C.

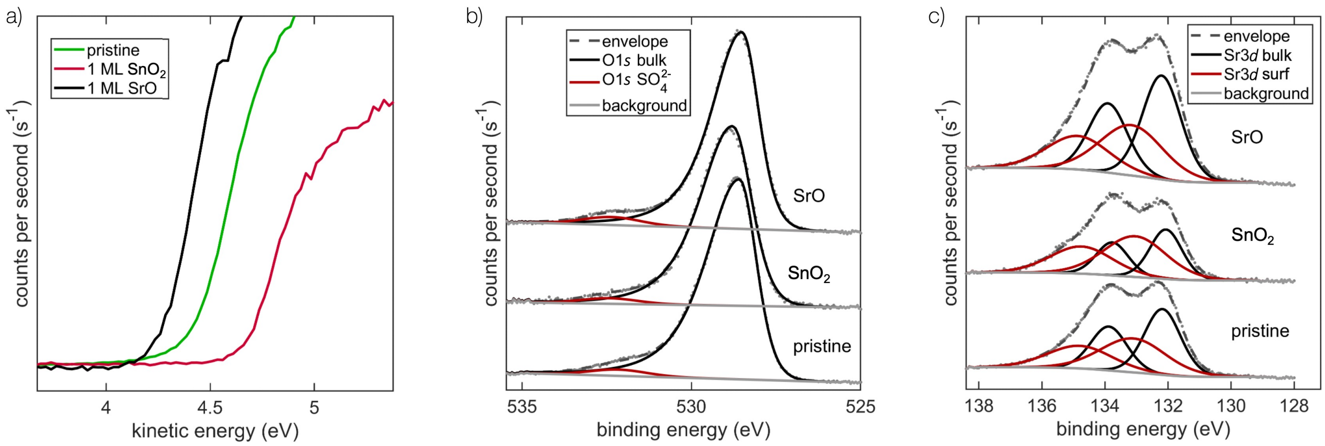

Supplementary Figure 2. a) low kinetic energy cutoff region for a pristine, a SrO decorated and a SnO_2 decorated 50 nm LSC thin film. b) O 1*s* region of a pristine, a SrO decorated and a SnO_2 decorated 50 nm LSC thin film. The region is fitted with two species, one for the main O 1*s* peak and one for a secondary peak which is related to SO_4^{2-}) traces on the thin film surface. c) Sr 3*d* region for a pristine, a SrO decorated and a SnO_2 decorated 50 nm LSC thin film.

The work function is highest on SnO_2 decorated LSC and lowest for SrO decorated LSC. The main oxygen 1*s* species exhibits some asymmetry which is attributed to the metal-like electronic structure of LSC (it has previously been shown that this peak asymmetry correlates with the metallicity of the electronic structure of perovskite oxides[12]). In addition, the main O 1*s* peak of SrO and SnO_2 decorated LSC is slightly broadened by the oxygen signature of the decoration, which is however not well distinguishable. The peak shape is further affected by the applied bias voltage (see below). Due to the strong overlap of the bulk and decorating oxide O 1*s* species, the peak asymmetry was optimized to match the envelope, rather than using two strongly covariant components.

When increasing the bias voltage, e.g. to 1000 mV (corresponding to $\approx$3.4 mbar), the peaks change slightly (see figure below for a measurement at 1.25 V). In particular, the work function of the SrO decorated LSC thin film increases more than for other surfaces and the previously SO_4^{2-} related species appears to grow. However, the sulphur signal does not change accordingly during this process. The combination of these phenomena leads us to believe that the growing peak is related to peroxide species whose presence on the surface can be tuned by the application of sample bias. This is also in accordance with computational results which show that the work function tends to increase upon peroxide formation. This phenomenon has not yet been investigated in detail, but may be the first indication of the spectroscopic observation of peroxide species which take part in the oxygen exchange mechanism. Critical to this approach is the fact that measurement are performed in UHV and also at relatively low temperatures (450 °C). Thereby, the surface exchange is very slow and anodic polarization leads to a very high oxygen chemical potential at the working electrode surface, facilitating the formation of these peroxide adsorbates. In addition, the XPS signature is visible in UHV, because no SO_4^{2-} adsorbates are present on the surface in these conditions. An in-depth exploration of this feature, however, goes beyond the scope of this study.

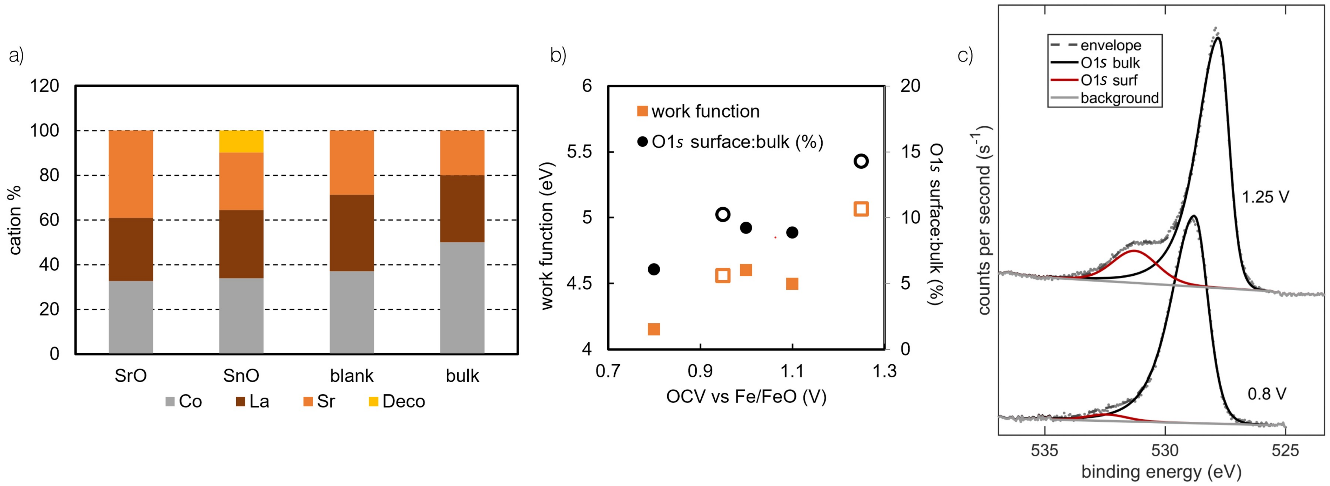

Supplementary Figure 3. a) Compositional quantification of the decorated LSC surface. 66 % of the signal stem from the topmost 1.6 nm. The SrO and SnO decoration account for roughly 10 atomic percent each – as much as expected for one monolayer. b) Work function (left axis) and O1s surface component area in percent of the O 1*s* bulk signal (right axis) of Sr decorated LSC in vacuum as function of the cell voltage acquired in UHV. Closed symbols were acquired at 450 °C, open symbols at 300 °C. Since the SO_4^{2-} coverage is constant (or at least not increasing) in UHV, the change of the O1*s* surface component area is possibly related to a SrO_2 termination forming at high anodic bias. c) O 1*s* region of a SrO decorated 50 nm LSC thin film with different applied bias voltages (0.8 and 1.25 V).

The following figure shows the Co 2*p* and the S 2*p* region of pristine and decorated thin films at 1000 mV polarization. Small amounts of sulphur are visible on the surface (the highest amount is observed on the SrO decorated film (5-10 % coverage) which is in line with more basic surfaces being more susceptible to acidic species). Deviations between the Co spectra are not clear enough to reliably determine any changes in oxidation states.

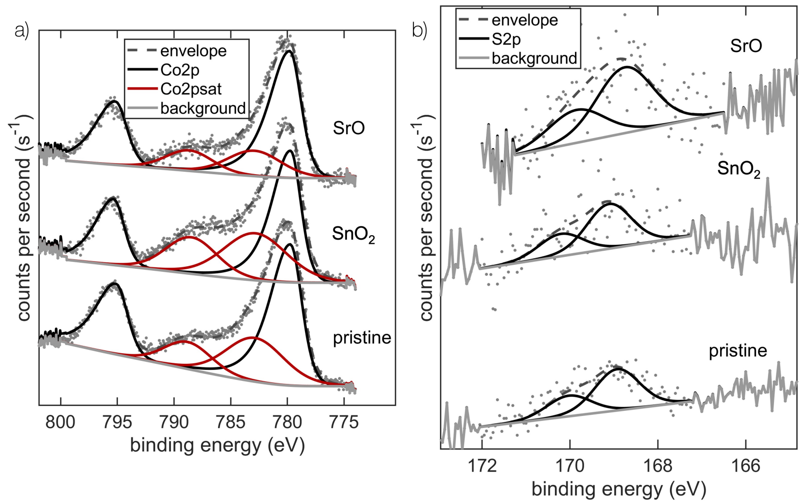

Supplementary Figure 4. a) Co 2*p* region of a pristine, a SrO decorated and a SnO_2 decorated 50 nm LSC thin film under 1000 mV polarization against a Fe/FeO electrode. b) S 2*p* region of a pristine, a SrO decorated and a SnO_2 decorated 50 nm LSC thin film under 1000 mV polarization against a Fe/FeO electrode.

Supplementary Note 3: Work function changes with calculated ionic potentials

While the main paper uses formal charges and crystal radii from Shannon[5] to determine the ionic potential of different surface decorations (or specifically surface cations) to emphasize the low-barrier access to a physically and chemically meaningful descriptor, it is also possible to determine ionic charge and ionic radius by a Bader charge analysis from DFT calculations. The correlation between the calculated work function and the calculated ionic potential is shown in the following figure:

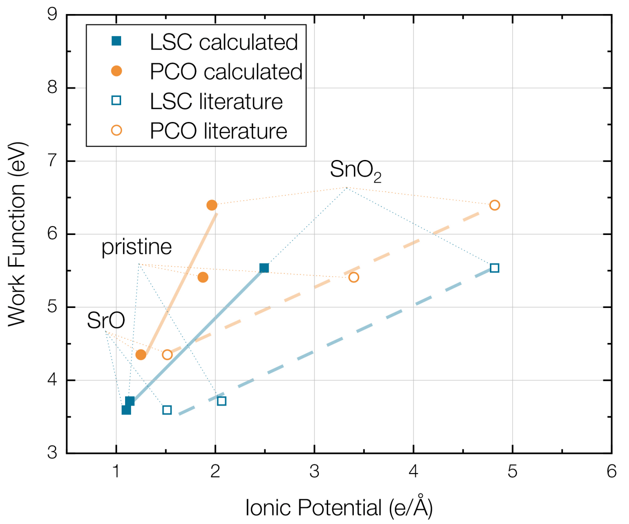

Supplementary Figure 5. Calculated work functions plotted against the ionic potential of the surface cation for ionic potentials resulting from DFT calculations and from literature ionic radii and formal charges.

The correlation still holds well with ionic potentials from DFT calculations, however, the slopes are much steeper than for ionic potentials estimated from formal charges and from literature ionic radii. In particular, it is not straightforward to evaluate ionic radii from Bader charges. In this case, we identified the ionic radius with the distance from a cation position to the zero-flux surface between the cation and the nearest surface oxygen atom.

Supplementary Note 4: Surface dipoles and chemical potentials

The redistribution of charge and the emergence of dipoles are closely linked to the chemical environment at the heterojunction, and especially to the chemical potentials of defects and charge carriers that may be involved in charge transfer processes. While the treatment of space charge zones as a consequence of standard chemical potential differences is a well-established practice for surfaces and grain boundaries in mixed conducting oxides, such as $SrTiO_3$[13–15], it also provides a foundation for charge redistribution in mixed conducting oxide heterojunctions. For instance, decorating LSC with an additional layer of SrO leads to an accumulation of electron density in the surface layer of LSC and to a depletion in the decoration layer and in the CoO_2 layer below. Regarding chemical potential differences, we suggest that changes in the decoration and surface are primarily induced by different chemical environments, while changes in the subsurface are triggered by short-range electrostatic effects. These effects are highly confined by the high charge carrier density and metal-like electronic structure of LSC. Comparing this system to simplified plate capacitors, the effects of charge redistribution (evaluated by a Bader charge analysis) result in a net potential increase of 0.11 V at the surface. This value precisely matches the decrease in work function suggested by DFT calculations on LSC decorated with one layer of SrO. Under more realistic conditions (particularly at high temperatures), similar chemical potential changes will also occur for oxygen vacancies, thereby adding complexity to the situation. It is noteworthy that standard space charge approaches such as Mott-Schottky or Gouy-Chapman models are not particularly suitable for the description of these phenomena due to the short range of electrostatic effects and discrete space-charge modelling approaches may be required for the investigation of defect concentrations in solid solution MIEC oxide heterojunctions[16].

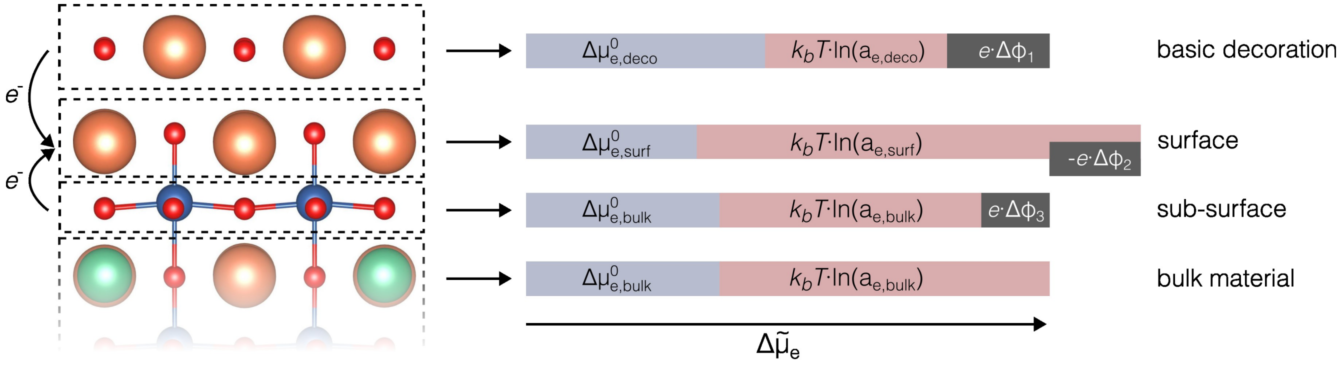

Supplementary Figure 6. Schematic of exemplary chemical potential changes upon surface decoration of LSC with SrO. The standard chemical potential increases in the decoration and decreases in the surface, leading to electron density redistribution and to space charge formation. Similar processes may occur for oxygen vacancies, leading to a more complicated chemical potential landscape. Other possibilities for surface dipole formation include geometric reconstructions such as surface buckling.

Supplementary Note 5: Pr density of states for decorated surfaces

Here, we aim to provide additional information about pristine surfaces after decoration but without the impact of oxygen adsorbates. While an in-depth analysis of surface decoration atoms themselves might be misleading, since both the exact atomic configuration, as well as the precise stoichiometry of the decoration are not known, we investigated a subsurface Pr atom below the decoration. In general, the results are as expected from a surface dipole perspective, with unoccupied Pr levels shifting towards the Fermi level for more basic surfaces. This analysis also showcases that charge redistribution likely not only concerns Pr as the redox active species but also oxygen atoms in the decoration and the subsurface. At this point it is important to emphasize that this analysis is only of limited use for realistic models, since at high temperatures, oxygen vacancy formation in both the decoration and the host material, as well as oxygen adsorbates will lead to a much more complicated charge redistribution between all participants.

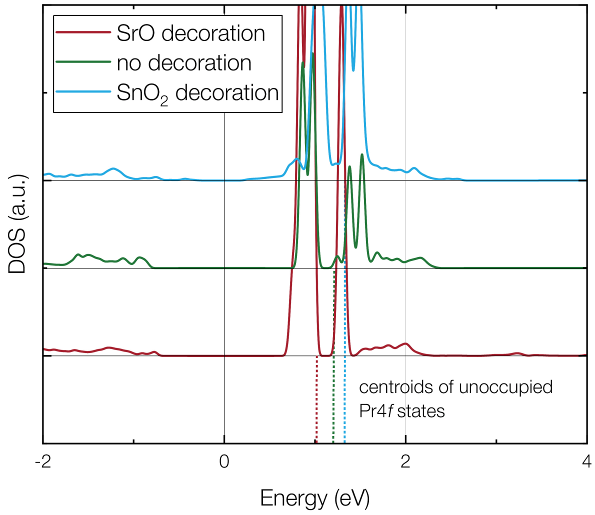

Supplementary Figure 7. Partial density of states of a Pr atom in the sub-surface for pure PCO and PCO decorated with SrO and SnO_2. In addition, the centroid of the unoccupied Pr4f states is shown, illustrating a shift of the Pr4*f* level to the Fermi level for more basic surfaces.

Supplementary Note 6: In-situ impedance spectroscopy during decoration

For a more in-depth discussion, we want to note here that detailed impedance spectroscopic investigations on thin films upon basic and acidic decoration have been presented by the authors in previous articles[11,17,18]. Generally, all i-PLD measurements follow a similar procedure, which has been outlined in previous studies[19]. The investigated samples consist of a YSZ single crystal electrolyte, Ti/Pt current collecting grids on both sides of the susbtrate and a 200 nm nanoporous LSC64 counterelectrode on one side. On the other side, the working electrode is grown during i-PLD. The temperature during deposition is controlled very precisely via the ohmic offset, which contains the electrolyte resistance (that is well known from literature[20]), as well as resistive contributions from wiring and the current collecting grids (that are measured beforehand). Resulting impedance spectra usually consist of three major contributions. i) the above mentioned ohmic offset, ii) a mid-frequency semicircle, which corresponds to the surface exchange resistance coupled with the chemical capacitance of the working electrode, and iii) a low-frequency arc, which corresponds to the surface exchange resistance coupled with the chemical capacitance of the counter electrode (which is only visible for low working electrode resistances). The volume-related chemical capacitance of the counter electrode is much higher due to the higher thickness, leading to different characteristic frequencies of the two electrode contributions, and thus to well separable semicircles. It is worth mentioning, that for some samples, a small high-frequency shoulder appears, which is attributed to interfacial resistances between thin film and electrolyte.

As the working electrode feature is usually a nearly perfect semicircle, it is very unlikely that diffusion limitations affect the measurements (they would be visible as Warburg-type distortions on the high frequency side of the semicircle) and thus, the observed resistance is directly connected to the rate of the rate determining step of the oxygen exchange reaction. Our previous results have suggested that this rate determining step is related to charge transfer and dissociation[21], but the details about this reaction mechanism are still not entirely clear. It is, however, very likely, that upstream reaction steps of the oxygen reduction reaction include adsorption of molecular oxygen and charge transfer onto the adsorbed oxygen.

During decoration, the only impedance contribution that is affected is the working electrode feature. Upon basic decoration, the surface exchange resistance decreases, suggesting faster oxygen exchange kinetics and *vice versa* for acidic decoration. In the figure below, exemplary impedance measurements of acidic and basic decoration for LSC64 and PCO are shown.

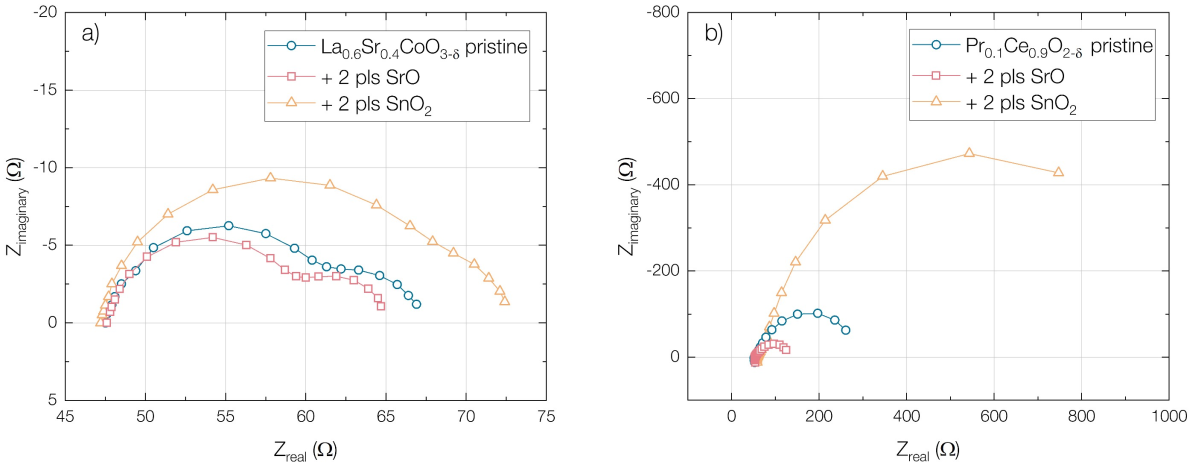

Supplementary Figure 8. a) Impedance spectra of $La_{0.6}Sr_{0.4}CoO_{3-\delta}$ in its pristine state as well as with 2 pls SrO and SnO_2 decoration. b) Impedance spectra of $Pr_{0.1}Ce_{0.9}O_{2-\delta}$ in its pristine state as well as with 2 pls SrO and SnO_2 decoration.

Supplementary Note 7: Deposition of thicker decoration layers

To evaluate the evolution of the oxygen exchange kinetics with growing thickness of a basic decoration layer, SrO was grown during i-PLD and the surface exchange resistance was tracked. Interestingly, the fastest kinetics were observed for decoration layers with a nominal thickness being slightly thinner than 1 monolayer (within experimental error, the optimal thickness is 1 monolayer). After that, the resistance starts to increase again. Mechanistically, we suggest that electronic interaction with the LSC bulk (which is essential for fast oxygen exchange) is still easy for one monolayer of SrO but gets increasingly difficult when depositing thicker layers. Preliminary results of a parallel study also suggest that the activation energy of the surface exchange resistance increases for thicker decoration layers, further supporting this hypothesis.

The same holds for PCO, where the fastest kinetics are reached at one monolayer. However, for PCO, the SrO layer can get relatively thick and still improve the kinetics of pristine PCO. This points towards the inherent differences between LSC and PCO, with LSC potentially being particularly active due to its electronic properties and easy electron transfer towards O_2 adsorbates.

More detailed studies of the activation energy of the oxygen exchange reaction with decoration thickness might be a viable opportunity to gain further insight into the underlying mechanism of the oxygen exchange reaction and are planned for future studies.

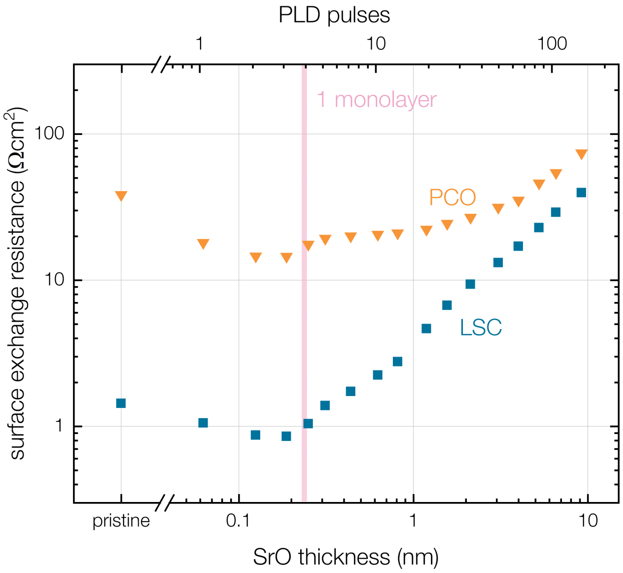

Supplementary Figure 9. Evolution of the surface exchange resistance of LSC and PCO with the thickness of a growing SrO decoration layer. In both cases, the kinetics reach their fastest value at a decoration layer thickness of ≈1 monolayer. Afterwards, the kinetics continuously decrease and the resistance increases correspondingly.

Supplementary Note 8: Extended sample characterization

In Fig. 10, atomic force microscopy (AFM) images of LSC and PCO surfaces with different decorations are shown. These measurements were performed for previous publications[11] and showcase that the decorations do not lead to any particle formation on the surface, or in fact to any visible alteration of the surface at all. For LSC, polycrystalline thin films with SnO_2 and CaO decorations were investigated. In both cases, the granular surface of the LSC thin film is completely unchanged and no visible traces of the decoration (nominally one unit cell) can be seen on the surface. In the case of PCO, the thin films were deposited on a YSZ/GDC system, leading to epitaxial thin film growth, with atomic terraces being visible in AFM. Again, both SnO_2 and CaO decorations do not agglomerate, and the very flat surface remains unchanged during decoration. Some isolated particles are found on the surfaces, which are attributed to dirt on the sample surface.

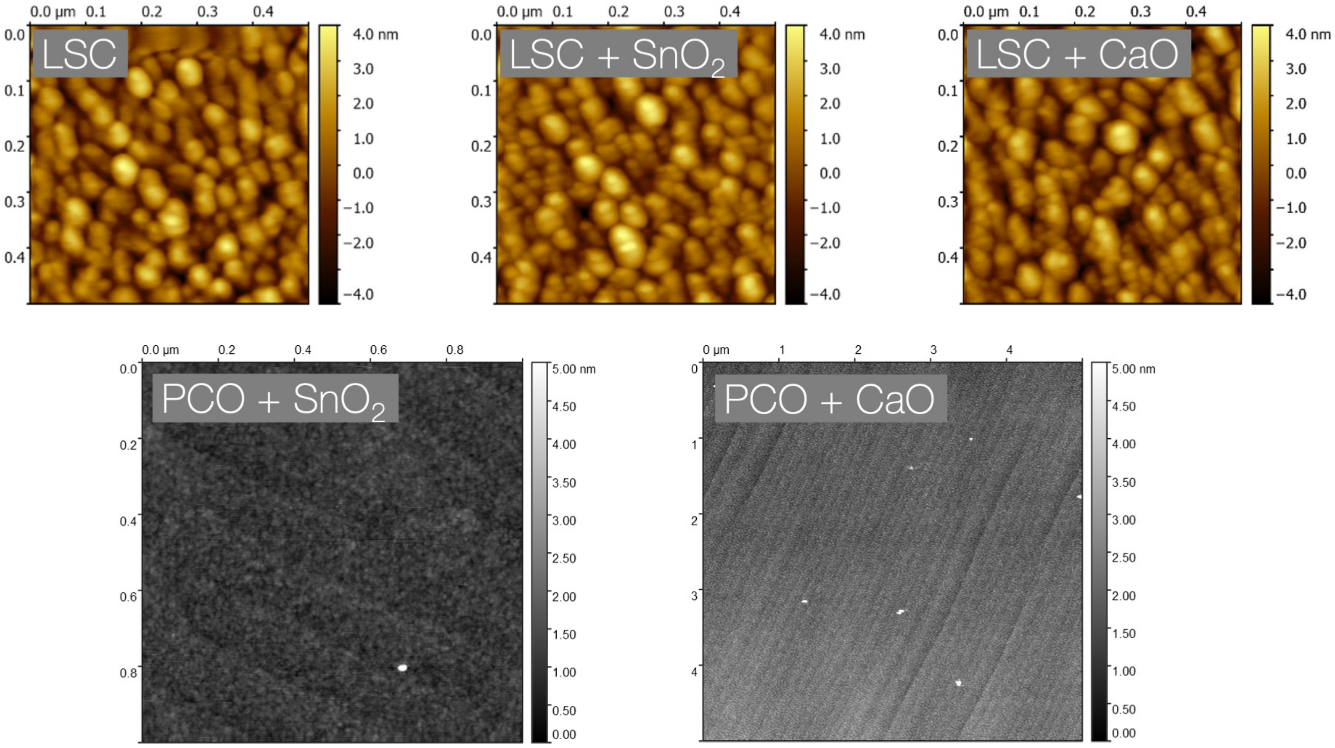

Supplementary Figure 10. AFM images of LSC and PCO surfaces upon decoration with SnO_2 and CaO. In both cases, no visible traces of the decoration can be seen in AFM images.

In addition to AFM measurements, low energy ion scattering (LEIS) measurements yield further insight into the chemistry of the outermost surface of PCO and LSC thin films. In a previous publication[11], LEIS was used to investigate PCO decorated with SnO_2 and SrO. For one nominal unit cell of SrO, no Ce or Pr signal could be identified during LEIS measurements, for SnO_2, the Sn signal amounts to around 90 % of the signal, indicating a high degree of coverage. For LSC, a SnO_2 (1 ML) decorated sample was investigated with a 5 keV $^{20}Ne^{+}$ primary analysis beam to allow for a proper separation of different cation signals. Again, the Sn signal is the largest signal contribution and dominates the surface cation chemistry (56 % of the total cation signal), however, the coverage does not seem to be as complete as for the case of PCO. Considering the low amounts of deposited material and the unchanged morphology during AFM measurements, the experimental evidence show that the decorations grow very flat and ultimately lead to full coverage, but they do not necessarily grow in a layer-by-layer growth mode. In the case of PCO, also depth profiles were recorded with LEIS[11], showing a sharp decrease of the decoration ion signal below the surface, showing no significant interdiffusion into the host material.

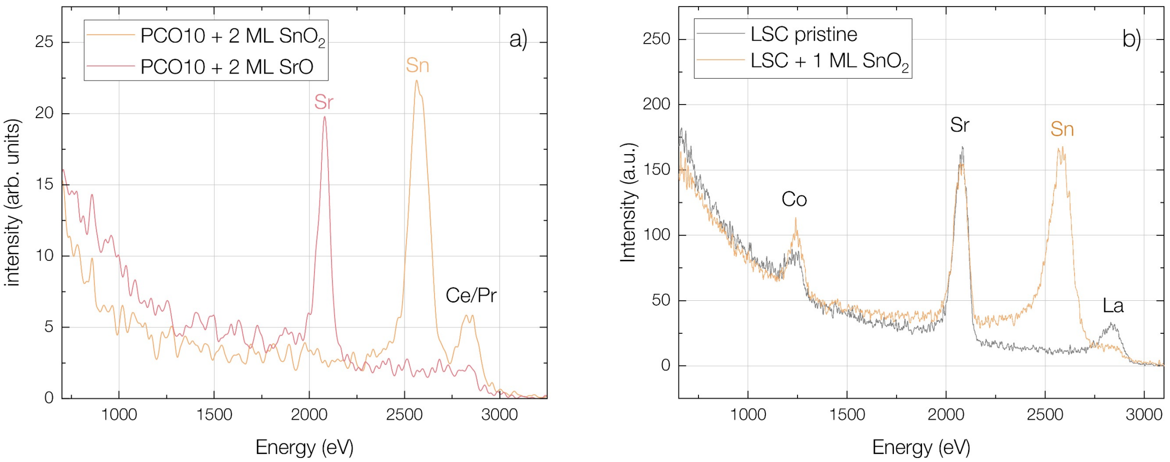

Supplementary Figure 11. LEIS spectra of a) PCO10 decorated with SnO_2 and SrO and b) LEIS spectra of pristine LSC and LSC decorated with 1 ML of SnO_2.

To combine chemical analysis with lateral resolution, we also performed a secondary ion mass spectrometry measurement on a 100 x 100 μm^2 area of SnO_2 decorated LSC. The measurement was performed in the collimated burst alignment (CBA) mode (10.1039/C3JA50059D) which allows for higher lateral resolution. Again, an overlay of the Sn and the Sr signal (probing depth around 1 nm) reveals no noticeable agglomeration in larger particles or islands.

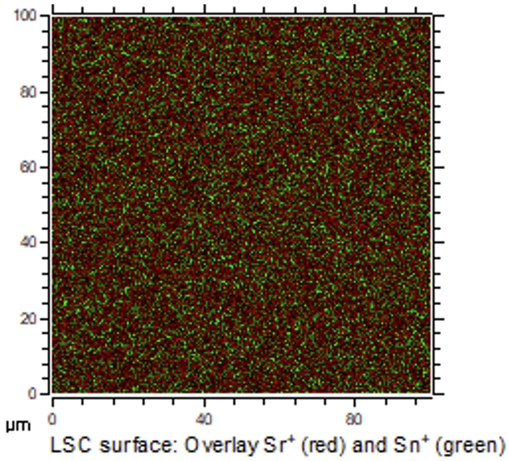

Supplementary Figure 12. Secondary ion mass spectrometry 2D map of SnO_2 decorated LSC in the CBA mode.

The combination of these results emphasizes the complexity of these surfaces, particularly at high temperatures, but also showcases that the decoration layers prefer a flat growth mode vs. island or particle growth. This also is reflected in the XPS results, which probe the average over the whole surface. The fact that XPS trends are not as pronounced as the corresponding DFT model calculations (in particular for LSC) might indicate that we indeed do not deal with a perfectly homogeneous monolayer coverage but with a somewhat nonideal coverage, i.e. only the majority of the surface is covered small amounts of the host material remain exposed at the surface.

Supplementary Note 9: DFT structures (unit cell dimensions and fractional coordinates)

LSC pristine

The host LSC structure is a 2x2x5 (001)-oriented cubic cell which has subsequently been relaxed. The lattice constant amounts to $\approx$ 3.82 Å. La atoms have been replaced with Sr atoms in a way that emulates Sr accumulation at the surface, which we would expect in high-temperature environments.

cell size (Å)	7.63338	7.63338	40.25053
atom	x	y	z
Sr1	0.99976	0.00019	0.26841
Sr2	0.00024	0.99981	0.73159
Sr3	0.49982	0.00012	0.26705
Sr4	0.50018	0.99988	0.73295
Sr5	0.99975	0.50017	0.26708
Sr6	0.00025	0.49983	0.73291
Sr7	0.49972	0.50019	0.26855
Sr8	0.50028	0.49981	0.73145
La1	0.99997	0.00005	0.45231
La2	0.00003	0.99995	0.54769
La3	0.49997	0.00001	0.45260
La4	0.50003	0.99999	0.54740
Sr9	0.99998	0.50000	0.45213
Sr10	0.00002	0.50000	0.54787
La5	0.49995	0.50002	0.45236
La6	0.50005	0.49998	0.54764
Sr11	0.00016	0.99989	0.64196
Sr12	0.99984	0.00011	0.35804
La7	0.50021	0.99984	0.64322
La8	0.49979	0.00016	0.35678
La9	0.00032	0.49972	0.64284
La10	0.99968	0.50028	0.35716
Sr13	0.50018	0.49986	0.64234
Sr14	0.49982	0.50014	0.35766
Co1	0.75131	0.75015	0.30759
Co2	0.24869	0.24985	0.69241
Co3	0.24940	0.74974	0.69228
Co4	0.75060	0.25026	0.30772
Co5	0.24818	0.25023	0.30760
Co6	0.75182	0.74977	0.69240
Co7	0.75101	0.24996	0.69229
Co8	0.24899	0.75004	0.30771
Co9	0.75187	0.74818	0.50000
Co10	0.24813	0.25182	0.50000
Co11	0.24796	0.74803	0.50000
Co12	0.75204	0.25197	0.50000
Co13	0.75032	0.74934	0.59555
Co14	0.24969	0.25066	0.40445
Co15	0.24957	0.74932	0.40447
Co16	0.75043	0.25068	0.59553
Co17	0.24981	0.25059	0.59554
Co18	0.75019	0.74941	0.40446
Co19	0.75024	0.25081	0.40446
Co20	0.24976	0.74919	0.59553

atom	x	y	z
O1	0.00003	0.74437	0.31256
O2	0.99997	0.25563	0.68744
O3	0.00041	0.74413	0.68734
O4	0.99959	0.25587	0.31266
O5	0.50009	0.75460	0.31023
O6	0.49991	0.24540	0.68977
O7	0.50048	0.75432	0.68987
O8	0.49952	0.24568	0.31013
O9	0.74421	0.00018	0.31127
O10	0.25579	0.99982	0.68873
O11	0.25542	0.00008	0.31119
O12	0.74458	0.99992	0.68881
O13	0.75552	0.50023	0.31149
O14	0.24448	0.49977	0.68851
O15	0.24409	0.50004	0.31157
O16	0.75591	0.49996	0.68843
O17	0.74471	0.00013	0.40689
O18	0.25529	0.99987	0.59311
O19	0.25519	0.99992	0.40695
O20	0.74481	0.00008	0.59305
O21	0.99985	0.75235	0.40649
O22	0.00015	0.24765	0.59351
O23	0.99994	0.75232	0.59359
O24	0.00006	0.24768	0.40641
O25	0.74725	0.50014	0.40681
O26	0.25275	0.49986	0.59319
O27	0.25273	0.49992	0.40676
O28	0.74727	0.50008	0.59324
O29	0.49985	0.75640	0.40717
O30	0.50015	0.24360	0.59283
O31	0.49993	0.75630	0.59275
O32	0.50007	0.24370	0.40725
O33	0.74449	0.75603	0.45298
O34	0.25551	0.24397	0.54702
O35	0.25477	0.75556	0.54703
O36	0.74523	0.24444	0.45297
O37	0.25528	0.24412	0.45298
O38	0.74472	0.75588	0.54702
O39	0.74504	0.24459	0.54703
O40	0.25496	0.75541	0.45297
O41	0.99986	0.76673	0.49999
O42	0.00014	0.23327	0.50002
O43	0.49984	0.75217	0.50002
O44	0.50016	0.24782	0.49998
O45	0.74723	0.00014	0.50001
O46	0.25277	0.99985	0.49999
O47	0.73367	0.50013	0.49999
O48	0.26633	0.49987	0.50001
O49	0.75618	0.75018	0.26136
O50	0.24382	0.24982	0.73865
O51	0.24601	0.74971	0.73855
O52	0.75399	0.25029	0.26145
O53	0.24286	0.25055	0.26136
O54	0.75714	0.74945	0.73864
O55	0.75417	0.25020	0.73856
O56	0.24583	0.74980	0.26144
O57	0.74558	0.75129	0.64162
O58	0.25442	0.24871	0.35838
O59	0.25339	0.75074	0.35836
O60	0.74661	0.24926	0.64164
O61	0.25371	0.24940	0.64162
O62	0.74629	0.75060	0.35838
O63	0.74590	0.24978	0.35836
O64	0.25410	0.75022	0.64164

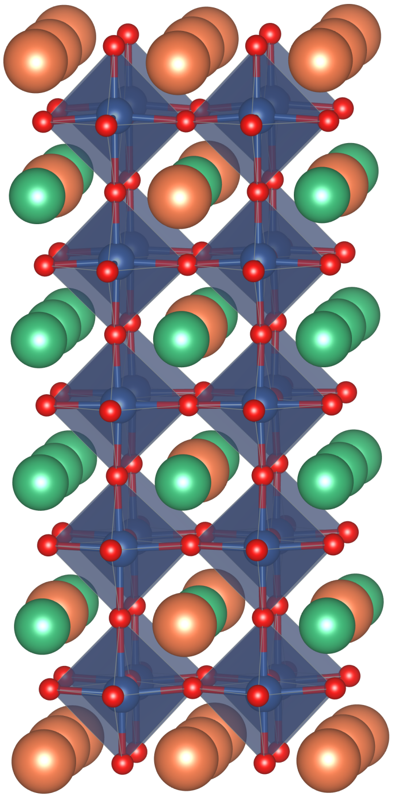

LSC with SrO decoration

The SrO decoration was placed on the LSC slab, continuing the SrO termination. This means that the first two layers correspont to a rock-salt structure with the [100] direction rotated by 45 ° compared to LSC. The Sr-Sr spacing in the decoration amounts along this direction amounts to 5.38 Å, compared to ≈ 5.16 Å in bulk SrO[22].

cell size (Å)	7.63338	7.63338	45.54230
atom	x	y	z
Sr1	0.49977	0.00017	0.29467
Sr2	0.50023	0.99983	0.70533
Sr3	0.99980	0.00013	0.29383
Sr4	0.00020	0.99986	0.70617
Sr5	0.49979	0.50015	0.29388
Sr6	0.50021	0.49985	0.70612
Sr7	0.99976	0.50017	0.29467
Sr8	0.00023	0.49983	0.70533
La1	0.49996	0.00008	0.45784
La2	0.50004	0.99992	0.54216
La3	0.99996	0.00002	0.45816
La4	0.00004	0.99998	0.54184
Sr9	0.49997	0.50000	0.45765
Sr10	0.50002	0.50000	0.54235
La5	0.99992	0.50003	0.45793
La6	0.00008	0.49997	0.54207
Sr11	0.50016	0.99988	0.62554
Sr12	0.49984	0.00012	0.37446
La7	0.00021	0.99984	0.62656
La8	0.99979	0.00016	0.37343
La9	0.50033	0.49972	0.62626
La10	0.49967	0.50028	0.37374
Sr13	0.00018	0.49986	0.62580
Sr14	0.99981	0.50014	0.37420
Co1	0.25048	0.75010	0.33066
Co2	0.74952	0.24990	0.66934
Co3	0.74965	0.74975	0.66935
Co4	0.25035	0.25025	0.33065
Co5	0.74906	0.25024	0.33066
Co6	0.25094	0.74976	0.66934
Co7	0.25075	0.24996	0.66935
Co8	0.74925	0.75004	0.33065
Co9	0.25200	0.74822	0.50000
Co10	0.74800	0.25178	0.50000
Co11	0.74796	0.74817	0.50000
Co12	0.25204	0.25183	0.50000
Co13	0.25035	0.74928	0.58429
Co14	0.74965	0.25072	0.41572
Co15	0.74959	0.74936	0.41572
Co16	0.25041	0.25063	0.58428
Co17	0.74980	0.25061	0.58428
Co18	0.25020	0.74939	0.41572
Co19	0.25019	0.25078	0.41572
Co20	0.74981	0.74922	0.58428
Sr15	0.25107	0.75025	0.23747
Sr16	0.74893	0.24974	0.76253
Sr17	0.75067	0.74975	0.76253
Sr18	0.24933	0.25025	0.23747
Sr19	0.74835	0.25017	0.23747
Sr20	0.25165	0.74983	0.76253
Sr21	0.24977	0.24995	0.76253
Sr22	0.75023	0.75005	0.23747

atom	x	y	z
O1	0.49987	0.74509	0.33477
O2	0.50013	0.25491	0.66523
O3	0.50023	0.74480	0.66515
O4	0.49977	0.25520	0.33485
O5	0.99987	0.75432	0.33339
O6	0.00013	0.24568	0.66661
O7	0.00023	0.75405	0.66669
O8	0.99977	0.24595	0.33331
O9	0.24536	0.00015	0.33402
O10	0.75464	0.99985	0.66598
O11	0.75426	0.00009	0.33396
O12	0.24574	0.99991	0.66604
O13	0.25569	0.50016	0.33418
O14	0.74431	0.49984	0.66582
O15	0.74391	0.50009	0.33424
O16	0.25609	0.49991	0.66576
O17	0.24502	0.00008	0.41781
O18	0.75498	0.99992	0.58219
O19	0.75480	0.00000	0.41789
O20	0.24520	1.00000	0.58210
O21	0.49991	0.75184	0.41728
O22	0.50009	0.24816	0.58272
O23	0.50003	0.75172	0.58284
O24	0.49997	0.24828	0.41717
O25	0.24691	0.50008	0.41776
O26	0.75309	0.49992	0.58224
O27	0.75301	0.50000	0.41768
O28	0.24699	0.50000	0.58232
O29	0.99991	0.75636	0.41818
O30	0.00009	0.24364	0.58182
O31	0.00002	0.75616	0.58171
O32	0.99998	0.24384	0.41829
O33	0.24512	0.75603	0.45846
O34	0.75488	0.24397	0.54154
O35	0.75423	0.75590	0.54154
O36	0.24577	0.24410	0.45846
O37	0.75439	0.24431	0.45847
O38	0.24561	0.75569	0.54154
O39	0.24530	0.24448	0.54154
O40	0.75470	0.75552	0.45847
O41	0.49996	0.76762	0.49996
O42	0.50004	0.23238	0.50004
O43	0.99995	0.75199	0.50004
O44	0.00005	0.24802	0.49996
O45	0.24652	0.00004	0.50003
O46	0.75348	0.99996	0.49997
O47	0.23354	0.50004	0.49997
O48	0.76646	0.49996	0.50003
O49	0.25280	0.75003	0.28919
O50	0.74720	0.24997	0.71081
O51	0.74750	0.74962	0.71081
O52	0.25250	0.25038	0.28919
O53	0.74646	0.25052	0.28920
O54	0.25354	0.74948	0.71080
O55	0.25271	0.25025	0.71081
O56	0.74729	0.74975	0.28919
O57	0.24540	0.75119	0.62501
O58	0.75460	0.24881	0.37499
O59	0.75362	0.75083	0.37499
O60	0.24638	0.24917	0.62501
O61	0.75370	0.24968	0.62501
O62	0.24630	0.75032	0.37499
O63	0.24552	0.24978	0.37499
O64	0.75448	0.75022	0.62501
O65	0.49975	0.00020	0.23906
O66	0.50025	0.99980	0.76094
O67	0.99981	0.00013	0.23844
O68	0.00019	0.99987	0.76156
O69	0.49977	0.50016	0.23826
O70	0.50023	0.49984	0.76174
O71	0.99972	0.50018	0.23925
O72	0.00028	0.49982	0.76074

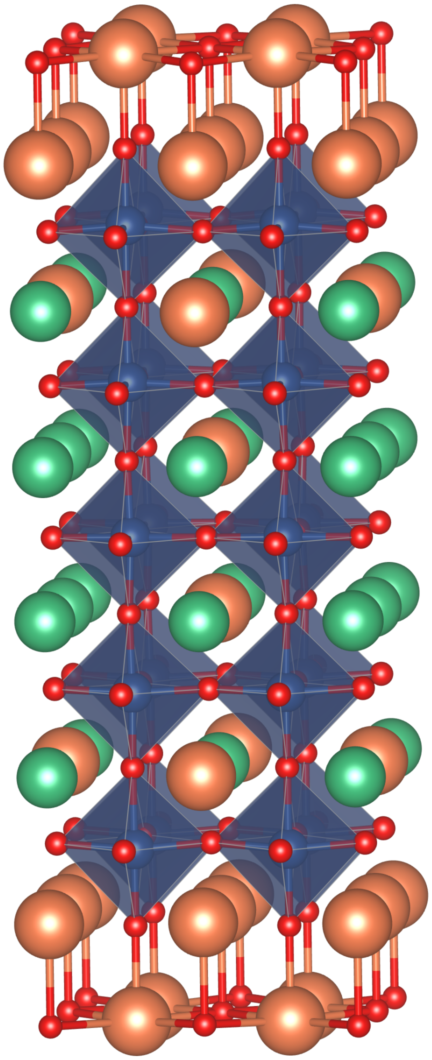

LSC with SnO_2 decoration

The SnO_2 decoration was placed on the LSC slab as a BO_2 perovskite layer, yielding one nominal unit cell of $SrSnO_3$ on the surface. The diagonal Sn-Sn distance amounts to 5.39 Å, compared to ≈ 5.72 Å in bulk $SrSnO_3$[23].

cell size (Å)	7.63338	7.63338	45.54230
atom	x	y	z
Sr1	0.49965	0.99893	0.29510
Sr2	0.50035	0.00107	0.70490
Sr3	0.99987	0.99973	0.29383
Sr4	0.00013	0.00027	0.70617
Sr5	0.50063	0.50044	0.29462
Sr6	0.49937	0.49956	0.70538
Sr7	0.99904	0.49986	0.29479
Sr8	0.00096	0.50014	0.70521
La1	0.49995	0.00000	0.45820
La2	0.50005	1.00000	0.54181
La3	0.99995	0.99999	0.45859
La4	0.00005	0.00001	0.54141
Sr9	0.49996	0.49986	0.45814
Sr10	0.50004	0.50014	0.54185
La5	0.99985	0.49991	0.45837
La6	0.00015	0.50009	0.54163
Sr11	0.50021	0.00032	0.62419
Sr12	0.49979	0.99968	0.37581
La7	0.00002	0.00023	0.62512
La8	0.99998	0.99976	0.37488
La9	0.50024	0.50025	0.62457
La10	0.49976	0.49975	0.37543
Sr13	0.00017	0.50033	0.62437
Sr14	0.99983	0.49967	0.37563
Co1	0.25007	0.74910	0.33375
Co2	0.74993	0.25090	0.66625
Co3	0.74957	0.74996	0.66629
Co4	0.25043	0.25004	0.33371
Co5	0.74926	0.24963	0.33375
Co6	0.25074	0.75037	0.66625
Co7	0.25047	0.25052	0.66620
Co8	0.74953	0.74948	0.33380
Co9	0.25251	0.74803	0.49999
Co10	0.74749	0.25197	0.50001
Co11	0.74749	0.74803	0.50000
Co12	0.25251	0.25197	0.50000
Co13	0.25052	0.74935	0.58319
Co14	0.74948	0.25065	0.41680
Co15	0.74947	0.74903	0.41682
Co16	0.25053	0.25097	0.58318
Co17	0.74966	0.25098	0.58318
Co18	0.25034	0.74902	0.41682
Co19	0.25031	0.25066	0.41680
Co20	0.74969	0.74934	0.58320
Sn1	0.25004	0.74904	0.24668
Sn2	0.74996	0.25096	0.75332
Sn3	0.74879	0.74968	0.75337
Sn4	0.25121	0.25032	0.24663
Sn5	0.74918	0.24972	0.24668
Sn6	0.25082	0.75028	0.75332
Sn7	0.25094	0.25046	0.75329
Sn8	0.74906	0.74954	0.24671

atom	x	y	z
O1	0.49990	0.74376	0.33631
O2	0.50010	0.25624	0.66369
O3	0.50014	0.74432	0.66347
O4	0.49986	0.25568	0.33653
O5	0.99990	0.75488	0.33497
O6	0.00010	0.24512	0.66503
O7	0.00013	0.75544	0.66533
O8	0.99987	0.24456	0.33467
O9	0.24446	0.99960	0.33535
O10	0.75554	0.00040	0.66465
O11	0.75540	0.99956	0.33547
O12	0.24460	0.00044	0.66452
O13	0.25733	0.49959	0.33591
O14	0.74267	0.50041	0.66408
O15	0.74246	0.49956	0.33587
O16	0.25754	0.50044	0.66413
O17	0.24572	0.99995	0.41834
O18	0.75428	0.00005	0.58166
O19	0.75393	0.99994	0.41852
O20	0.24607	0.00006	0.58148
O21	0.49993	0.75023	0.41764
O22	0.50007	0.24977	0.58236
O23	0.50008	0.74978	0.58265
O24	0.49992	0.25022	0.41735
O25	0.24609	0.49993	0.41841
O26	0.75391	0.50007	0.58159
O27	0.75365	0.49992	0.41824
O28	0.24635	0.50008	0.58176
O29	0.99994	0.75710	0.41887
O30	0.00006	0.24290	0.58113
O31	0.00007	0.75677	0.58091
O32	0.99993	0.24323	0.41910
O33	0.24537	0.75687	0.45875
O34	0.75463	0.24313	0.54125
O35	0.75334	0.75701	0.54126
O36	0.24666	0.24299	0.45874
O37	0.75337	0.24398	0.45875
O38	0.24663	0.75602	0.54125
O39	0.24533	0.24414	0.54125
O40	0.75467	0.75586	0.45875
O41	0.50000	0.76879	0.49989
O42	0.50000	0.23121	0.50011
O43	0.00000	0.75027	0.50012
O44	1.00000	0.24973	0.49988
O45	0.24525	0.00000	0.50008
O46	0.75475	1.00000	0.49992
O47	0.23516	0.50000	0.49992
O48	0.76484	0.50000	0.50008
O49	0.25271	0.74663	0.29218
O50	0.74729	0.25337	0.70782
O51	0.74501	0.74830	0.70791
O52	0.25499	0.25170	0.29209
O53	0.74489	0.24970	0.29218
O54	0.25511	0.75030	0.70782
O55	0.25328	0.25146	0.70773
O56	0.74672	0.74854	0.29227
O57	0.24392	0.75163	0.62389
O58	0.75608	0.24837	0.37611
O59	0.75461	0.75108	0.37614
O60	0.24539	0.24892	0.62386
O61	0.75443	0.24968	0.62388
O62	0.24557	0.75032	0.37612
O63	0.24393	0.24922	0.37609
O64	0.75607	0.75078	0.62392
O65	0.00025	0.24817	0.24435
O66	0.99975	0.75182	0.75565
O67	0.50017	0.24785	0.24147
O68	0.49983	0.75215	0.75853
O69	0.24829	0.49973	0.24200
O70	0.75171	0.50027	0.75800
O71	0.24905	0.99954	0.24400
O72	0.75095	0.00046	0.75600
O73	0.49967	0.74924	0.24309
O74	0.50033	0.25076	0.75691
O75	0.99945	0.74892	0.24449
O76	0.00055	0.25109	0.75551
O77	0.75137	0.49953	0.24371
O78	0.24863	0.50047	0.75629
O79	0.75107	0.99965	0.24384
O80	0.24893	0.00035	0.75616

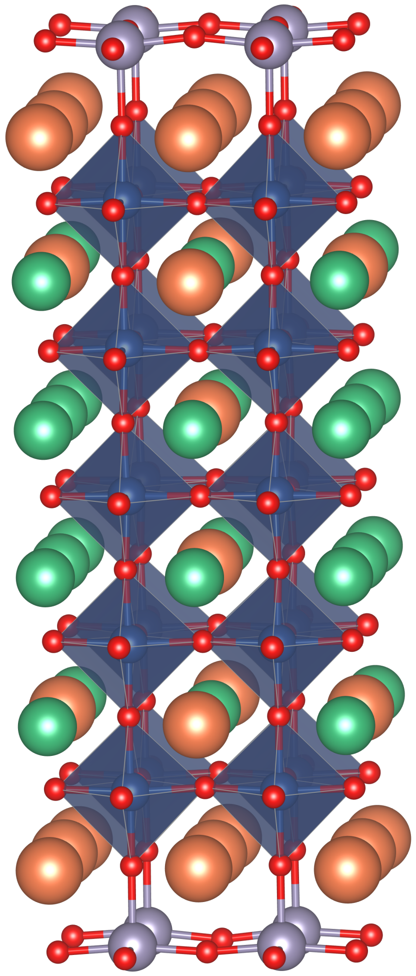

LSC with SO_3 adsorbates

Two SO_3 adsorbates were placed diagonally on the LSC slab surface in a tetrahedral configuration. S-O bond lengths are 1.46 Å in the SO_3 unit and 1.65 Å to the surface oxygen atom, compared to 1.49 Å in a SO_4^{2-} anion[24].

cell size	7.63338	7.63338	45.54230
atom	x	y	z
Sr1	0.99957	0.00021	0.26351
Sr2	0.00043	0.99979	0.73649
Sr3	0.49976	0.00014	0.26381
Sr4	0.50024	0.99986	0.73619
Sr5	0.99961	0.50031	0.26325
Sr6	0.00039	0.49969	0.73675
Sr7	0.49966	0.50026	0.26448
Sr8	0.50034	0.49974	0.73552
La1	0.99997	0.00002	0.45242
La2	0.00003	0.99998	0.54758
La3	0.49998	0.00002	0.45240
La4	0.50002	0.99998	0.54760
Sr9	0.99997	0.50002	0.45208
Sr10	0.00003	0.49998	0.54792
La5	0.49998	0.50002	0.45238
La6	0.50002	0.49998	0.54762
Sr11	0.00015	0.99990	0.64222
Sr12	0.99985	0.00010	0.35778
La7	0.50020	0.99982	0.64366
La8	0.49980	0.00018	0.35634
La9	0.00022	0.49981	0.64361
La10	0.99978	0.50019	0.35639
Sr13	0.50014	0.49992	0.64255
Sr14	0.49986	0.50008	0.35745
Co1	0.75012	0.75042	0.31125
Co2	0.24988	0.24958	0.68875
Co3	0.24845	0.74996	0.69112
Co4	0.75155	0.25004	0.30888
Co5	0.24931	0.24992	0.31123
Co6	0.75069	0.75008	0.68877
Co7	0.75203	0.24975	0.69112
Co8	0.24797	0.75025	0.30888
Co9	0.75262	0.74735	0.50000
Co10	0.24738	0.25265	0.50000
Co11	0.24812	0.74802	0.50000
Co12	0.75188	0.25198	0.50000
Co13	0.75131	0.74864	0.59493
Co14	0.24869	0.25136	0.40507
Co15	0.24924	0.74934	0.40459
Co16	0.75076	0.25066	0.59541
Co17	0.24892	0.25119	0.59491
Co18	0.75108	0.74881	0.40508
Co19	0.75056	0.25081	0.40459
Co20	0.24944	0.74919	0.59541
S1	0.73492	0.75064	0.22077
S2	0.26508	0.24937	0.77923
S3	0.26373	0.25027	0.22079
S4	0.73627	0.74973	0.77921

atom	x	y	z
O1	0.99985	0.74271	0.31272
O2	0.00015	0.25729	0.68728
O3	0.00023	0.74263	0.68717
O4	0.99977	0.25737	0.31283
O5	0.50013	0.75548	0.31057
O6	0.49987	0.24452	0.68943
O7	0.50049	0.75533	0.68955
O8	0.49951	0.24467	0.31045
O9	0.74121	0.00011	0.31143
O10	0.25879	0.99989	0.68857
O11	0.25860	0.00014	0.31133
O12	0.74140	0.99986	0.68867
O13	0.75455	0.50025	0.31165
O14	0.24544	0.49975	0.68836
O15	0.24528	0.49996	0.31174
O16	0.75472	0.50004	0.68826
O17	0.74442	0.00008	0.40683
O18	0.25558	0.99992	0.59317
O19	0.25557	0.99998	0.40684
O20	0.74443	0.00002	0.59316
O21	0.99987	0.75328	0.40655
O22	0.00013	0.24672	0.59345
O23	0.99996	0.75330	0.59346
O24	0.00004	0.24670	0.40654
O25	0.74653	0.50012	0.40660
O26	0.25347	0.49988	0.59340
O27	0.25348	0.49994	0.40659
O28	0.74651	0.50006	0.59341
O29	0.49990	0.75606	0.40685
O30	0.50010	0.24394	0.59315
O31	0.49997	0.75606	0.59313
O32	0.50003	0.24394	0.40687
O33	0.74302	0.75705	0.45276
O34	0.25698	0.24295	0.54724
O35	0.25521	0.75539	0.54710
O36	0.74479	0.24461	0.45290
O37	0.25696	0.24296	0.45276
O38	0.74304	0.75704	0.54725
O39	0.74477	0.24463	0.54710
O40	0.25523	0.75537	0.45290
O41	0.99993	0.76578	0.50000
O42	0.00007	0.23422	0.50000
O43	0.50006	0.75201	0.50000
O44	0.49994	0.24799	0.50000
O45	0.74778	0.99994	0.50000
O46	0.25222	0.00006	0.50000
O47	0.73435	0.50007	0.50000
O48	0.26565	0.49993	0.50000
O49	0.76635	0.75009	0.26125
O50	0.23365	0.24991	0.73875
O51	0.24365	0.74980	0.73549
O52	0.75635	0.25020	0.26451
O53	0.23229	0.25095	0.26127
O54	0.76771	0.74905	0.73873
O55	0.75662	0.25005	0.73549
O56	0.24338	0.74995	0.26451
O57	0.74714	0.75143	0.64219
O58	0.25286	0.24857	0.35781
O59	0.25167	0.75080	0.35864
O60	0.74833	0.24920	0.64136
O61	0.25234	0.24904	0.64218
O62	0.74766	0.75096	0.35782
O63	0.74795	0.24947	0.35864
O64	0.25205	0.75053	0.64136
O65	0.54110	0.74961	0.21772
O66	0.45890	0.25039	0.78228
O67	0.54245	0.74892	0.78222
O68	0.45755	0.25109	0.21778
O69	0.81783	0.91546	0.21039
O70	0.18217	0.08454	0.78961
O71	0.81881	0.91481	0.78956
O72	0.18119	0.08519	0.21044
O73	0.81835	0.58668	0.21016
O74	0.18166	0.41332	0.78984
O75	0.81976	0.58600	0.78992
O76	0.18024	0.41400	0.21008

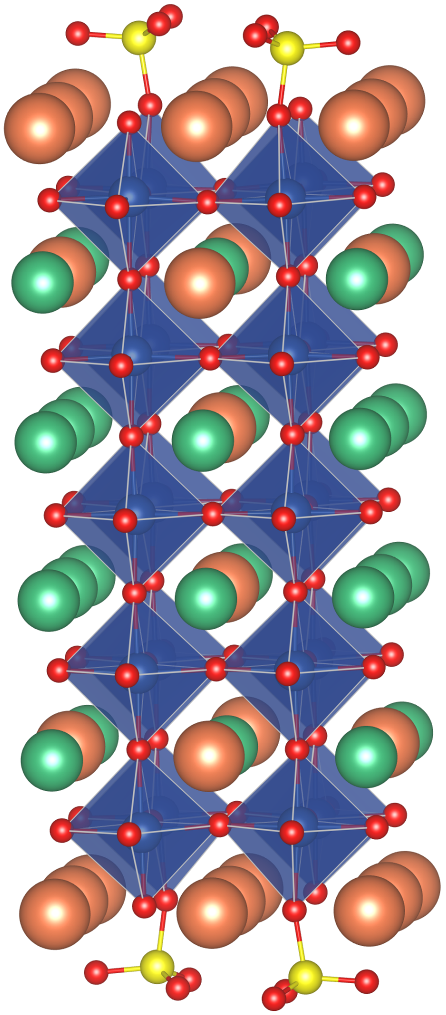

PCO pristine

The PCO structure is a 2x2x2 (111)-oriented and hexagonal cell with $\gamma = 120°$ and a cubic lattice parameter of 5.44 Å. One Ce atom in the surface and one Ce atom in the center were replaced with Pr to emulate a PCO10 stoichiometry.

cell size (Å)	7.73220	7.73220	40.10688
atom	x	y	z
Ce1	0.83330	0.16670	0.34241
Ce2	0.16670	0.83330	0.65759
Ce3	0.83298	0.66652	0.34229
Ce4	0.66652	0.83298	0.65771
Ce5	0.33348	0.16702	0.34229
Ce6	0.16702	0.33348	0.65771
Ce7	0.00023	0.99977	0.73547
Ce8	0.99977	0.00023	0.26453
Ce9	0.50012	0.00064	0.73546
Ce10	0.00064	0.50012	0.26454
Ce11	0.99936	0.49988	0.73546
Ce12	0.49988	0.99936	0.26454
Ce13	0.00000	0.00000	0.50000
Ce14	0.50000	0.00000	0.50000
Ce15	0.00000	0.50000	0.50000
Ce16	0.33331	0.16684	0.57867
Ce17	0.16684	0.33331	0.42133
Ce18	0.83316	0.66669	0.57867
Ce19	0.66669	0.83316	0.42133
Ce20	0.83331	0.16669	0.57881
Ce21	0.16669	0.83331	0.42119
Ce22	0.66640	0.33360	0.42128
Ce23	0.33360	0.66640	0.57872
Ce24	0.33389	0.66611	0.34239
Ce25	0.66611	0.33389	0.65761
Pr1	0.49948	0.50052	0.73548
Pr2	0.50052	0.49948	0.26452
Pr3	0.50000	0.50000	0.50000

atom	x	y	z
O1	0.33327	0.16724	0.51969
O2	0.16724	0.33327	0.48031
O3	0.83276	0.66672	0.51969
O4	0.66672	0.83276	0.48031
O5	0.83310	0.16690	0.51958
O6	0.16690	0.83310	0.48042
O7	0.66551	0.33449	0.48038
O8	0.33449	0.66551	0.51962
O9	0.33341	0.16706	0.28433
O10	0.16706	0.33341	0.71567
O11	0.83294	0.66659	0.28433
O12	0.66659	0.83294	0.71567
O13	0.83292	0.16708	0.28430
O14	0.16708	0.83292	0.71570
O15	0.66487	0.33513	0.71576
O16	0.33513	0.66487	0.28424
O17	0.16674	0.33339	0.59841
O18	0.33339	0.16674	0.40159
O19	0.66661	0.83326	0.59841
O20	0.83326	0.66661	0.40159
O21	0.16673	0.83327	0.59841
O22	0.83327	0.16673	0.40159
O23	0.33339	0.66661	0.40159
O24	0.66661	0.33339	0.59841
O25	0.16637	0.33318	0.36203
O26	0.33318	0.16637	0.63797
O27	0.66682	0.83363	0.36203
O28	0.83363	0.66682	0.63797
O29	0.16676	0.83324	0.36203
O30	0.83324	0.16676	0.63797
O31	0.33316	0.66684	0.63800
O32	0.66684	0.33316	0.36200
O33	0.00010	0.99990	0.67781
O34	0.99990	0.00010	0.32219
O35	0.50045	0.00059	0.67787
O36	0.00059	0.50045	0.32213
O37	0.99941	0.49955	0.67787
O38	0.49955	0.99941	0.32213
O39	0.49971	0.50029	0.67744
O40	0.50029	0.49971	0.32256
O41	0.00010	0.99990	0.44098
O42	0.99990	0.00010	0.55902
O43	0.50029	0.00023	0.44104
O44	0.00023	0.50029	0.55896
O45	0.99977	0.49971	0.44104
O46	0.49971	0.99977	0.55896
O47	0.49986	0.50014	0.44100
O48	0.50014	0.49986	0.55900
O49	0.33266	0.16566	0.75475
O50	0.16566	0.33266	0.24525
O51	0.83434	0.66734	0.75475
O52	0.66734	0.83434	0.24525
O53	0.83268	0.16732	0.75463
O54	0.16732	0.83268	0.24537
O55	0.66476	0.33524	0.24521
O56	0.33524	0.66476	0.75479

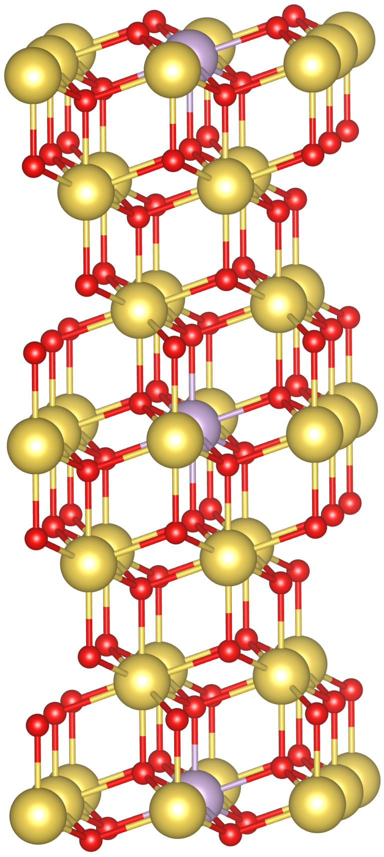

PCO with SnO_2 decoration

For SnO_2-decorated PCO, The fluorite structure was continued for one layer with Sn as the main cation. This leads to a shortest Sn-O distance of 2.32 Å, compared to 2.16 Å for a fluorite SnO_2 structure[25].

cell size (Å)	7.73220	7.73220	45.39864
atom	x	y	z
Ce1	0.83341	0.16659	0.36261
Ce2	0.16659	0.83341	0.63739
Ce3	0.83345	0.66660	0.36248
Ce4	0.66660	0.83345	0.63752
Ce5	0.33340	0.16655	0.36248
Ce6	0.16655	0.33340	0.63752
Ce7	0.00006	0.99994	0.70580
Ce8	0.99994	0.00006	0.29420
Ce9	0.49999	0.00003	0.70578
Ce10	0.00003	0.49999	0.29422
Ce11	0.99997	0.50001	0.70578
Ce12	0.50001	0.99997	0.29422
Ce13	0.00000	0.00000	0.50000
Ce14	0.50000	0.00000	0.50000
Ce15	0.00000	0.50000	0.50000
Ce16	0.33341	0.16701	0.56885
Ce17	0.16701	0.33341	0.43115
Ce18	0.83299	0.66659	0.56885
Ce19	0.66659	0.83299	0.43115
Ce20	0.83327	0.16673	0.56890
Ce21	0.16673	0.83327	0.43110
Ce22	0.66658	0.33342	0.43117
Ce23	0.33342	0.66658	0.56883
Ce24	0.33314	0.66686	0.36245
Ce25	0.66686	0.33314	0.63755
Pr1	0.49995	0.50005	0.70587
Pr2	0.50005	0.49995	0.29413
Pr3	0.50000	0.50000	0.50000
Sn1	0.66727	0.33273	0.22947
Sn2	0.33273	0.66727	0.77053
Sn3	0.83336	0.16664	0.77046
Sn4	0.16664	0.83336	0.22954
Sn5	0.33347	0.16647	0.77054
Sn6	0.16647	0.33347	0.22946
Sn7	0.83353	0.66653	0.77054
Sn8	0.66653	0.83353	0.22946

atom	x	y	z
O1	0.33368	0.16753	0.51710
O2	0.16753	0.33368	0.48290
O3	0.83247	0.66632	0.51710
O4	0.66632	0.83247	0.48290
O5	0.83329	0.16671	0.51712
O6	0.16671	0.83329	0.48288
O7	0.66631	0.33369	0.48285
O8	0.33369	0.66631	0.51714
O9	0.33358	0.16614	0.31102
O10	0.16614	0.33358	0.68898
O11	0.83386	0.66642	0.31102
O12	0.66642	0.83386	0.68898
O13	0.83366	0.16634	0.31137
O14	0.16634	0.83366	0.68863
O15	0.66809	0.33191	0.68884
O16	0.33191	0.66809	0.31116
O17	0.16677	0.33350	0.58601
O18	0.33350	0.16677	0.41399
O19	0.66650	0.83323	0.58601
O20	0.83323	0.66650	0.41399
O21	0.16660	0.83340	0.58604
O22	0.83340	0.16660	0.41396
O23	0.33345	0.66655	0.41399
O24	0.66655	0.33345	0.58601
O25	0.16765	0.33365	0.37951
O26	0.33365	0.16765	0.62049
O27	0.66635	0.83235	0.37951
O28	0.83235	0.66635	0.62049
O29	0.16663	0.83337	0.37957
O30	0.83337	0.16663	0.62043
O31	0.33379	0.66621	0.62046
O32	0.66621	0.33379	0.37954
O33	0.99975	0.00025	0.65440
O34	0.00025	0.99975	0.34560
O35	0.49984	0.99974	0.65430
O36	0.99974	0.49984	0.34570
O37	0.00026	0.50016	0.65430
O38	0.50016	0.00026	0.34570
O39	0.50010	0.49990	0.65508
O40	0.49990	0.50010	0.34492
O41	0.99999	0.00001	0.44820
O42	0.00001	0.99999	0.55180
O43	0.50022	0.99995	0.44820
O44	0.99995	0.50022	0.55180
O45	0.00005	0.49978	0.44820
O46	0.49978	0.00005	0.55180
O47	0.50010	0.49990	0.44839
O48	0.49990	0.50010	0.55161
O49	0.33336	0.16552	0.72350
O50	0.16552	0.33336	0.27650
O51	0.83448	0.66664	0.72350
O52	0.66664	0.83448	0.27650
O53	0.83365	0.16635	0.72366
O54	0.16635	0.83365	0.27634
O55	0.66867	0.33133	0.27639
O56	0.33133	0.66867	0.72361
O57	0.66478	0.33522	0.78548
O58	0.33522	0.66478	0.21452
O59	0.33454	0.16950	0.21459
O60	0.16950	0.33454	0.78541
O61	0.83051	0.66546	0.21459
O62	0.66546	0.83051	0.78541
O63	0.83316	0.16684	0.21451
O64	0.16684	0.83316	0.78549
O65	0.50134	0.00113	0.75687
O66	0.00113	0.50134	0.24313
O67	0.99887	0.49866	0.75687
O68	0.49866	0.99887	0.24313
O69	0.49964	0.50036	0.75562
O70	0.50036	0.49964	0.24438
O71	0.00000	1.00000	0.75675
O72	1.00000	0.00000	0.24325

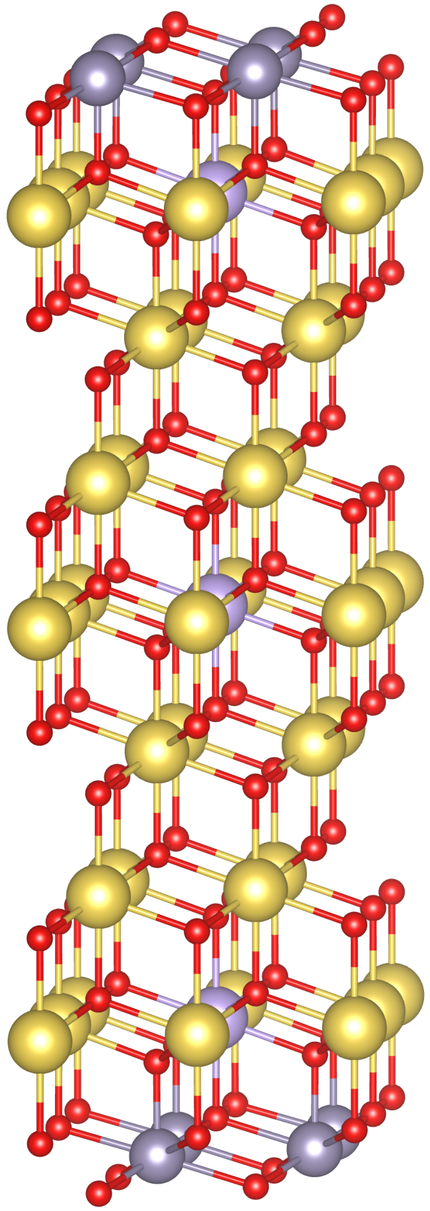

PCO with SrO decoration

The SrO decoration was placed hexagonally on top of the PCO slab, with the O atoms placed above the Sr layer to emulate a (111) SrO layer. During relaxation, the oxygen atoms moved towards the PCO bulk, yielding a largely Sr terminated surface with a very low work function. However, this structure proved to be energetically unfavorable and a SrO_2 termination was identified as a more stable structure. The shortest Sr-Sr distance amounts to 3.86 Å vs. 3.65 Å in SrO bulk[22].

cell size	7.73220	7.73220	45.39864
atom	x	y	z
Ce1	0.83344	0.16656	0.36227
Ce2	0.16656	0.83344	0.63773
Ce3	0.83320	0.66647	0.36211
Ce4	0.66647	0.83320	0.63789
Ce5	0.33353	0.16680	0.36211
Ce6	0.16680	0.33353	0.63789
Ce7	0.00010	0.99990	0.70625
Ce8	0.99990	0.00010	0.29375
Ce9	0.49952	0.99962	0.70618
Ce10	0.99962	0.49952	0.29382
Ce11	0.00038	0.50048	0.70618
Ce12	0.50048	0.00038	0.29382
Ce13	0.00000	0.00000	0.50000
Ce14	0.50000	0.00000	0.50000
Ce15	0.00000	0.50000	0.50000
Ce16	0.33351	0.16721	0.56888
Ce17	0.16721	0.33351	0.43112
Ce18	0.83279	0.66649	0.56888
Ce19	0.66649	0.83279	0.43112
Ce20	0.83323	0.16677	0.56904
Ce21	0.16677	0.83323	0.43096
Ce22	0.66652	0.33348	0.43115
Ce23	0.33348	0.66652	0.56885
Ce24	0.33322	0.66678	0.36203
Ce25	0.66678	0.33322	0.63797
Pr1	0.49996	0.50004	0.70620
Pr2	0.50004	0.49996	0.29380
Pr3	0.50000	0.50000	0.50000
Sr1	0.66627	0.33373	0.76690
Sr2	0.33373	0.66627	0.23310
Sr3	0.83276	0.16724	0.23235
Sr4	0.16724	0.83276	0.76765
Sr5	0.33297	0.16599	0.23295
Sr6	0.16599	0.33297	0.76705
Sr7	0.83401	0.66703	0.23295
Sr8	0.66703	0.83401	0.76705

atom	x	y	z
O1	0.33447	0.16899	0.51698
O2	0.16899	0.33447	0.48302
O3	0.83101	0.66553	0.51698
O4	0.66553	0.83101	0.48302
O5	0.83328	0.16672	0.51710
O6	0.16672	0.83328	0.48290
O7	0.66569	0.33431	0.48298
O8	0.33431	0.66569	0.51702
O9	0.33427	0.16725	0.31047
O10	0.16725	0.33427	0.68953
O11	0.83275	0.66573	0.31047
O12	0.66573	0.83275	0.68953
O13	0.83380	0.16620	0.31085
O14	0.16620	0.83380	0.68915
O15	0.66810	0.33190	0.68934
O16	0.33190	0.66810	0.31066
O17	0.16715	0.33370	0.58622
O18	0.33370	0.16715	0.41378
O19	0.66630	0.83285	0.58622
O20	0.83285	0.66630	0.41378
O21	0.16660	0.83340	0.58621
O22	0.83340	0.16660	0.41379
O23	0.33368	0.66632	0.41377
O24	0.66632	0.33368	0.58623
O25	0.16721	0.33340	0.37930
O26	0.33340	0.16721	0.62070
O27	0.66660	0.83279	0.37930
O28	0.83279	0.66660	0.62070
O29	0.16662	0.83338	0.37929
O30	0.83338	0.16662	0.62071
O31	0.33361	0.66639	0.62067
O32	0.66639	0.33361	0.37933
O33	0.99963	0.00037	0.65502
O34	0.00037	0.99963	0.34498
O35	0.49981	0.99958	0.65489
O36	0.99958	0.49981	0.34511
O37	0.00042	0.50019	0.65489
O38	0.50019	0.00042	0.34511
O39	0.50016	0.49984	0.65541
O40	0.49984	0.50016	0.34459
O41	0.99982	0.00018	0.44814
O42	0.00018	0.99982	0.55186
O43	0.50054	0.00008	0.44813
O44	0.00008	0.50054	0.55187
O45	0.99992	0.49946	0.44813
O46	0.49946	0.99992	0.55187
O47	0.50011	0.49989	0.44869
O48	0.49989	0.50011	0.55131
O49	0.33420	0.16699	0.72284
O50	0.16699	0.33420	0.27716
O51	0.83301	0.66580	0.72284
O52	0.66580	0.83301	0.27716
O53	0.83394	0.16606	0.72317
O54	0.16606	0.83394	0.27683
O55	0.66868	0.33132	0.27694
O56	0.33132	0.66868	0.72306
O57	0.49947	0.50053	0.76284
O58	0.50053	0.49947	0.23716
O59	0.50016	0.00039	0.76396
O60	0.00039	0.50016	0.23604
O61	0.99961	0.49984	0.76396
O62	0.49984	0.99961	0.23604
O63	0.00060	0.99940	0.76364
O64	0.99940	0.00060	0.23636

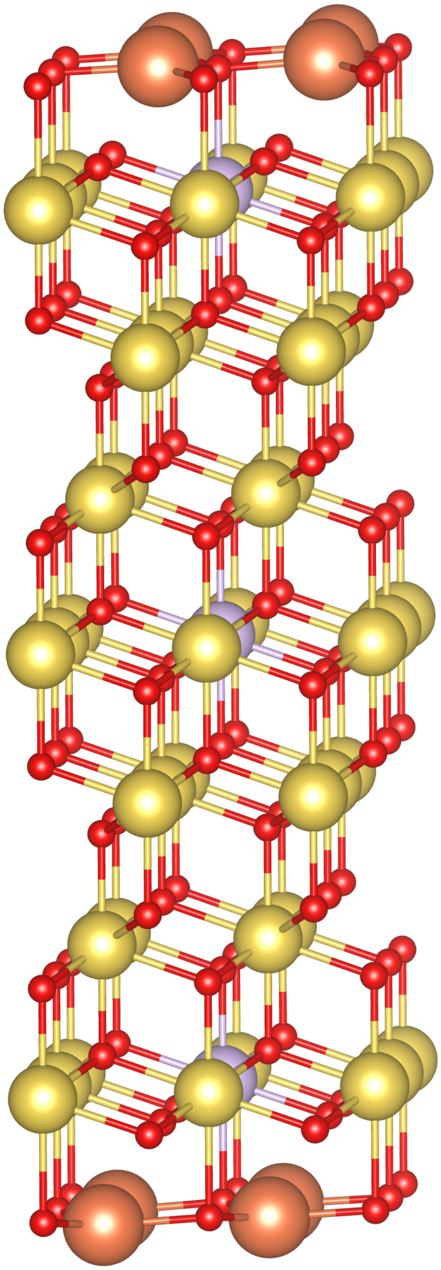

PCO with SrO_2 decoration

For the SrO_2 decoration, additional O atoms were placed on top of the relaxed O atoms in the SrO decoration. Up to a full SrO_2 layer, this proved to be energetically favorable. This also increased the work function considerably towards more resasonable values (with a better agreement with experimental values). The O-O bond length in the peroxide amounts to 1.50 Å, compared to 1.45-1.48 Å for bulk SrO_2[26].

cell size	7.73220	7.73220	45.39864
atom	x	y	z
Ce1	0.83344	0.16656	0.36249
Ce2	0.16656	0.83344	0.63751
Ce3	0.83232	0.66626	0.36255
Ce4	0.66626	0.83232	0.63745
Ce5	0.33374	0.16768	0.36255
Ce6	0.16768	0.33374	0.63745
Ce7	0.99963	0.00037	0.70596
Ce8	0.00037	0.99963	0.29405
Ce9	0.49995	0.99951	0.70585
Ce10	0.99951	0.49995	0.29415
Ce11	0.00049	0.50005	0.70585
Ce12	0.50005	0.00049	0.29415
Ce13	0.00000	0.00000	0.50000
Ce14	0.50000	0.00000	0.50000
Ce15	0.00000	0.50000	0.50000
Ce16	0.33326	0.16650	0.56864
Ce17	0.16650	0.33326	0.43136
Ce18	0.83350	0.66674	0.56864
Ce19	0.66674	0.83350	0.43136
Ce20	0.83329	0.16671	0.56873
Ce21	0.16671	0.83329	0.43127
Ce22	0.66675	0.33325	0.43139
Ce23	0.33325	0.66675	0.56861
Ce24	0.33334	0.66666	0.36244
Ce25	0.66666	0.33334	0.63756
Pr1	0.49956	0.50044	0.70586
Pr2	0.50044	0.49956	0.29414
Pr3	0.50000	0.50000	0.50000
Sr1	0.66320	0.33680	0.77688
Sr2	0.33680	0.66320	0.22312
Sr3	0.83094	0.16906	0.22113
Sr4	0.16906	0.83094	0.77887
Sr5	0.33086	0.16416	0.22119
Sr6	0.16416	0.33086	0.77881
Sr7	0.83584	0.66914	0.22119
Sr8	0.66914	0.83584	0.77881

atom	x	y	z
O1	0.33321	0.16660	0.51701
O2	0.16660	0.33321	0.48299
O3	0.83340	0.66679	0.51701
O4	0.66679	0.83340	0.48299
O5	0.83335	0.16665	0.51720
O6	0.16665	0.83335	0.48280
O7	0.66681	0.33319	0.48294
O8	0.33319	0.66681	0.51706
O9	0.33547	0.16840	0.31139
O10	0.16840	0.33547	0.68861
O11	0.83160	0.66453	0.31139
O12	0.66453	0.83160	0.68861
O13	0.83405	0.16595	0.31080
O14	0.16595	0.83405	0.68919
O15	0.66833	0.33167	0.68875
O16	0.33167	0.66833	0.31125
O17	0.16721	0.33367	0.58575
O18	0.33367	0.16721	0.41425
O19	0.66633	0.83279	0.58575
O20	0.83279	0.66633	0.41425
O21	0.16686	0.83314	0.58583
O22	0.83314	0.16686	0.41417
O23	0.33367	0.66633	0.41418
O24	0.66633	0.33367	0.58583
O25	0.16568	0.33258	0.37967
O26	0.33258	0.16568	0.62033
O27	0.66742	0.83432	0.37967
O28	0.83432	0.66742	0.62033
O29	0.16655	0.83346	0.37961
O30	0.83346	0.16655	0.62039
O31	0.33344	0.66656	0.62036
O32	0.66656	0.33344	0.37964
O33	0.99987	0.00013	0.65504
O34	0.00013	0.99987	0.34496
O35	0.49877	0.99913	0.65490
O36	0.99913	0.49877	0.34510
O37	0.00087	0.50123	0.65490
O38	0.50123	0.00087	0.34510
O39	0.50118	0.49882	0.65435
O40	0.49882	0.50118	0.34565
O41	0.99984	0.00016	0.44836
O42	0.00016	0.99984	0.55164
O43	0.50012	0.99981	0.44838
O44	0.99981	0.50012	0.55162
O45	0.00019	0.49988	0.44838
O46	0.49988	0.00019	0.55162
O47	0.50009	0.49991	0.44885
O48	0.49991	0.50009	0.55115
O49	0.33650	0.17234	0.72258
O50	0.17234	0.33650	0.27742
O51	0.82766	0.66350	0.72258
O52	0.66350	0.82766	0.27742
O53	0.83575	0.16425	0.72252
O54	0.16425	0.83575	0.27748
O55	0.66743	0.33257	0.27738
O56	0.33257	0.66743	0.72262
O57	0.49537	0.50463	0.76350
O58	0.50463	0.49537	0.23650
O59	0.50135	0.00078	0.76295
O60	0.00078	0.50135	0.23705
O61	0.99922	0.49865	0.76295
O62	0.49865	0.99922	0.23705
O63	0.00277	0.99723	0.76330
O64	0.99723	0.00277	0.23670
O65	0.49839	0.50161	0.79666
O66	0.50161	0.49839	0.20334
O67	0.00253	0.99747	0.79645
O68	0.99747	0.00253	0.20355
O69	0.50348	0.00548	0.79609
O70	0.00548	0.50348	0.20391
O71	0.99452	0.49652	0.79609
O72	0.49652	0.99452	0.20391
O71	0.99452	0.49652	0.79609
O72	0.49652	0.99452	0.20391

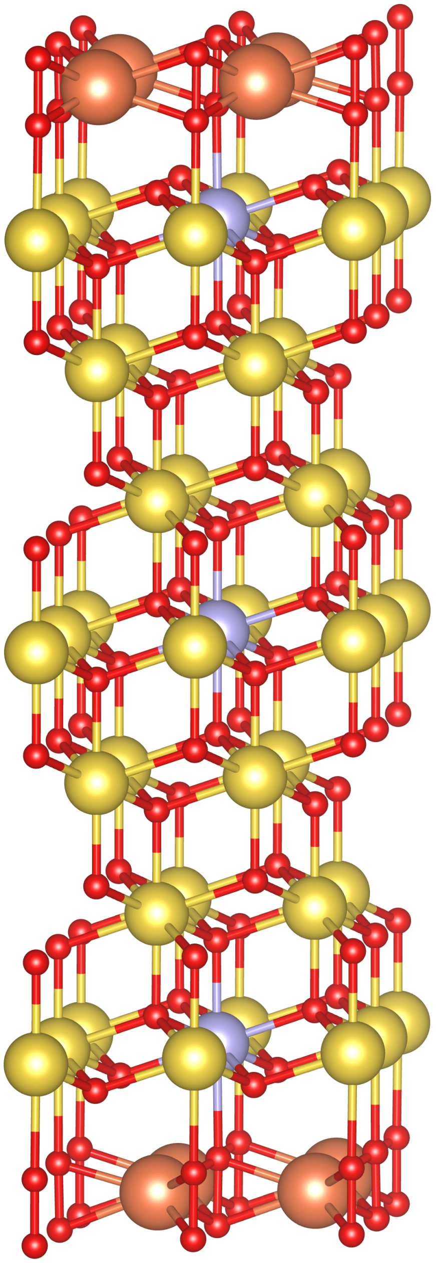

PCO with SO_3 adsorbates

Two SO_3 adsorbates were placed on two diagonally spaced top oxygen atoms of the PCO surface. S-O bond lengths are 1.47 Å in the SO_3 unit and 1.62 Å to the surface oxygen atom, compared to 1.49 Å in a SO_4^{2-} anion[24].

cell size	7.73220	7.73220	45.39864
atom	x	y	z
Ce1	0.83360	0.16640	0.36355
Ce2	0.16640	0.83360	0.63645
Ce3	0.83315	0.66501	0.36155
Ce4	0.66501	0.83315	0.63845
Ce5	0.33499	0.16685	0.36155
Ce6	0.16685	0.33499	0.63845
Ce7	0.00579	0.99421	0.70582
Ce8	0.99421	0.00579	0.29418
Ce9	0.49156	0.00664	0.70517
Ce10	0.00664	0.49156	0.29483
Ce11	0.99336	0.50844	0.70517
Ce12	0.50844	0.99336	0.29483
Ce13	0.00000	0.00000	0.50000
Ce14	0.50000	0.00000	0.50000
Ce15	0.00000	0.50000	0.50000
Ce16	0.33269	0.16795	0.56862
Ce17	0.16795	0.33269	0.43138
Ce18	0.83205	0.66731	0.56862
Ce19	0.66731	0.83205	0.43138
Ce20	0.83329	0.16671	0.56903
Ce21	0.16671	0.83329	0.43097
Ce22	0.66653	0.33347	0.43131
Ce23	0.33347	0.66653	0.56869
Ce24	0.33532	0.66468	0.36272
Ce25	0.66468	0.33532	0.63728
Pr1	0.50557	0.49443	0.70957
Pr2	0.49443	0.50557	0.29043
Pr3	0.50000	0.50000	0.50000
S1	0.84300	0.15700	0.76429
S2	0.15700	0.84300	0.23572
S3	0.67834	0.32166	0.24020
S4	0.32166	0.67834	0.75980

atom	x	y	z
O1	0.33338	0.16752	0.51707
O2	0.16752	0.33338	0.48293
O3	0.83248	0.66662	0.51707
O4	0.66662	0.83248	0.48293
O5	0.83345	0.16655	0.51714
O6	0.16655	0.83345	0.48287
O7	0.66651	0.33349	0.48288
O8	0.33349	0.66651	0.51712
O9	0.33109	0.14614	0.31013
O10	0.14614	0.33109	0.68987
O11	0.85386	0.66891	0.31013
O12	0.66891	0.85386	0.68987
O13	0.82643	0.17357	0.31267
O14	0.17357	0.82643	0.68733
O15	0.67517	0.32483	0.68960
O16	0.32483	0.67517	0.31040
O17	0.16633	0.33492	0.58603
O18	0.33492	0.16633	0.41397
O19	0.66508	0.83367	0.58603
O20	0.83367	0.66508	0.41397
O21	0.16676	0.83324	0.58548
O22	0.83324	0.16676	0.41452
O23	0.33377	0.66623	0.41401
O24	0.66623	0.33377	0.58599
O25	0.16848	0.33057	0.37990
O26	0.33057	0.16848	0.62010
O27	0.66943	0.83152	0.37990
O28	0.83152	0.66943	0.62010
O29	0.16557	0.83442	0.37903
O30	0.83442	0.16557	0.62097
O31	0.33356	0.66644	0.62084
O32	0.66644	0.33356	0.37916
O33	0.00260	0.99740	0.65574
O34	0.99740	0.00260	0.34427
O35	0.49805	0.00492	0.65405
O36	0.00492	0.49805	0.34595
O37	0.99508	0.50195	0.65405
O38	0.50195	0.99508	0.34595
O39	0.50064	0.49936	0.65591
O40	0.49936	0.50064	0.34409
O41	0.00040	0.99960	0.44829
O42	0.99960	0.00040	0.55171
O43	0.49985	0.99916	0.44836
O44	0.99916	0.49985	0.55164
O45	0.00084	0.50015	0.44836
O46	0.50015	0.00084	0.55164
O47	0.50037	0.49962	0.44866
O48	0.49962	0.50037	0.55134
O49	0.32296	0.14797	0.72056
O50	0.14797	0.32296	0.27944
O51	0.85203	0.67704	0.72056
O52	0.67704	0.85203	0.27944
O53	0.83025	0.16975	0.72927
O54	0.16975	0.83025	0.27073
O55	0.67022	0.32978	0.27579
O56	0.32978	0.67022	0.72421
O57	0.74448	0.25552	0.77647
O58	0.25552	0.74448	0.22353
O59	0.76431	0.94155	0.77026
O60	0.23569	0.05845	0.22974
O61	0.05845	0.23569	0.77026
O62	0.94155	0.76431	0.22974
O63	0.57461	0.42539	0.23104
O64	0.42539	0.57461	0.76896
O65	0.58142	0.10547	0.23465
O66	0.41858	0.89453	0.76535
O67	0.89453	0.41858	0.23465
O68	0.10547	0.58142	0.76535

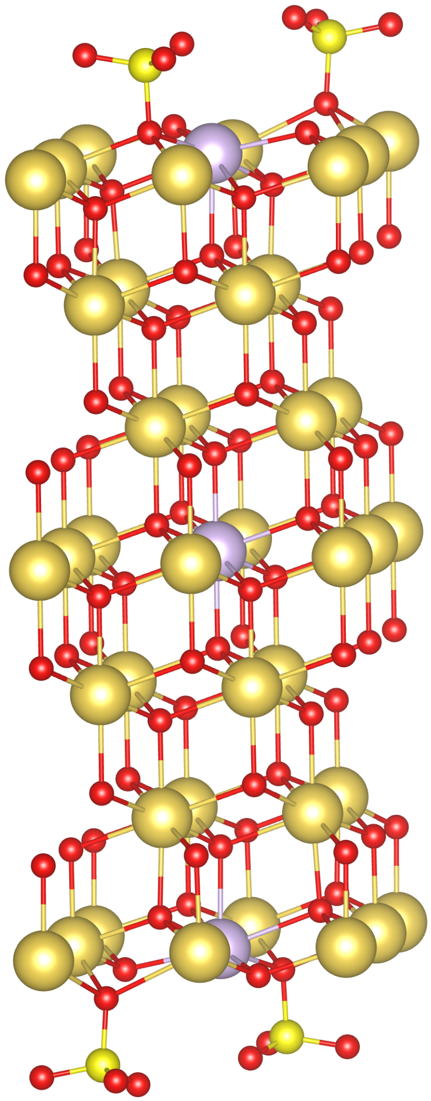

Supplementary References

1. Cartledge, G. The Correlation of Thermochemical Data by the Ionic Potential. *The Journal of Physical Chemistry* **55,** 248–256 (1951).
2. Cartledge, G. Studies on the periodic system. I. The ionic potential as a periodic function1. *Journal of the American Chemical Society* **50,** 2855–2863 (1928).
3. Railsback, L. B. An earth scientist's periodic table of the elements and their ions. *Geology* **31,** 737–740 (2003).
4. Li, R., Yang, W., Su, Y., Li, Q., Gao, S. & Shang, J. K. Ionic potential: a general material criterion for the selection of highly efficient arsenic adsorbents. *Journal of Materials Science & Technology* **30,** 949–953 (2014).
5. Shannon, R. D. Revised effective ionic radii and systematic studies of interatomic distances in halides and chalcogenides. *Acta crystallographica section A: crystal physics, diffraction, theoretical and general crystallography* **32,** 751–767 (1976).
6. Smith, D. W. An acidity scale for binary oxides. *Journal of Chemical Education* **64,** 480 (1987).
7. Matar, S. F., Campet, G. & Subramanian, M. A. Electronic properties of oxides: Chemical and theoretical approaches. *Progress in Solid State Chemistry* **39,** 70–95 (2011).
8. Bratsch, S. G. Electronegativity and the acid-base character of binary oxides. *Journal of Chemical Education* **65,** 877 (1988).
9. Li, K. & Xue, D. Estimation of electronegativity values of elements in different valence states. *The Journal of Physical Chemistry A* **110,** 11332–11337 (2006).
10. Zhuravlev, V. Calculation of the bond ionicity in complex oxides. *Bulletin of the Russian Academy of Sciences. Physics* **71,** 681 (2007).
11. Riedl, C., Siebenhofer, M., Nenning, A., Wilson, G. E., Kilner, J., Rameshan, C., Limbeck, A., Opitz, A. K., Kubicek, M. & Fleig, J. Surface Decorations on Mixed Ionic and Electronic Conductors: Effects on Surface Potential, Defects, and the Oxygen Exchange Kinetics. *ACS Applied Materials & Interfaces* **15,** 26787–26798 (2023).
12. Nenning, A., Opitz, A. K., Rameshan, C., Rameshan, R., Blume, R., Hävecker, M., Knop-Gericke, A., Rupprechter, G., Klötzer, B. & Fleig, J. Ambient pressure XPS study of mixed conducting perovskite-type SOFC cathode and anode materials under well-defined electrochemical polarization. *The Journal of Physical Chemistry C* **120,** 1461–1471 (2016).
13. De Souza, R. A. & Martin, M. Using $^{18}O/^{16}O$ exchange to probe an equilibrium space-charge layer at the surface of a crystalline oxide: method and application. *Physical Chemistry Chemical Physics* **10,** 2356–2367 (2008).
14. De Souza, R. A. The formation of equilibrium space-charge zones at grain boundaries in the perovskite oxide $SrTiO_3$. *Physical Chemistry Chemical Physics* **11,** 9939–9969 (2009).
15. De Souza, R. A., Fleig, J., Merkle, R. & Maier, J. $SrTiO_3$: a model electroceramic. *International Journal of Materials Research* **94,** 218–225 (2022).
16. Xiao, C., Chen, C.-C. & Maier, J. Discrete modeling of ionic space charge zones in solids. *Physical Chemistry Chemical Physics* **24,** 11945–11957 (2022).
17. Siebenhofer, M., Riedl, C., Nenning, A., Artner, W., Rameshan, C., Opitz, A. K., Fleig, J. & Kubicek, M. Improving and degrading the oxygen exchange kinetics of $La_{0.6}Sr_{0.4}CoO_{3-\delta}$ by Sr decoration. *Journal of Materials Chemistry A* **11,** 12827–12836 (2023).

18. Rupp, G. M., Opitz, A. K., Nenning, A., Limbeck, A. & Fleig, J. Real-time impedance monitoring of oxygen reduction during surface modification of thin film cathodes. *Nature materials* **16,** 640–645 (2017).

19. Siebenhofer, M., Huber, T. M., Friedbacher, G., Artner, W., Fleig, J. & Kubicek, M. Oxygen exchange kinetics and nonstoichiometry of pristine $La_{0.6}Sr_{0.4}CoO_{3-\delta}$ thin films unaltered by degradation. *Journal of Materials Chemistry A* **8,** 7968–7979 (2020).

20. Ahamer, C., Opitz, A. K., Rupp, G. & Fleig, J. Revisiting the temperature dependent ionic conductivity of yttria stabilized zirconia (YSZ). *Journal of the Electrochemical Society* **164,** F790 (2017).

21. Siebenhofer, M., Riedl, C., Schmid, A., Limbeck, A., Opitz, A. K., Fleig, J. & Kubicek, M. Investigating oxygen reduction pathways on pristine SOFC cathode surfaces by in situ PLD impedance spectroscopy. *Journal of Materials Chemistry A* **10,** 2305–2319 (2022).

22. Copeland, W. & Swalin, R. Studies on the defect structure of strontium oxide. *Journal of Physics and Chemistry of Solids* **29,** 313–325 (1968).

23. Mohan, T., Kuppusamy, S. & Michael, R. J. V. Tuning of Structural and Magnetic Properties of $SrSnO_3$ Nanorods in Fabrication of Blocking Layers for Enhanced Performance of Dye-Sensitized Solar Cells. *ACS omega* **7,** 18531–18541 (2022).

24. Yan, C., Zhu, L., Dong, J., Gu, D., Jiang, H. & Wang, B. Structural modification of isomorphous SO_4^{2-}-doped K_2FeO_4 for remediating the stability and enhancing the discharge of super-iron battery. *Royal Society Open Science* **6,** 180919 (2019).

25. Das, S. & Jayaraman, V. SnO_2: A comprehensive review on structures and gas sensors. *Progress in Materials Science* **66,** 112–255 (2014).

26. Königstein, M. Structural properties of nonstoichiometric barium and strontium peroxides: BaO_{2-x} ($1.97 \geq$ 2-x ≥ 1.72) and SrO_{2-x} ($1.98 \geq$ 2-x ≥ 1.90). *Journal of Solid State Chemistry* **147,** 478–484 (1999).
